# Development and Validation of a Biodynamic Model for Mechanistically Predicting Metal Accumulation in Fish-Parasite Systems

**DOI:** 10.1371/journal.pone.0161091

**Published:** 2016-08-22

**Authors:** T. T. Yen Le, Milen Nachev, Daniel Grabner, A. Jan Hendriks, Bernd Sures

**Affiliations:** 1 Department of Aquatic Ecology and Centre for Water and Environmental Research (ZWU), University of Duisburg-Essen, Essen, Germany; 2 Department of Environmental Science, Faculty of Science, Radboud University Nijmegen, Nijmegen, the Netherlands; 3 Department of Zoology, University of Johannesburg, PO Box 524, Auckland Park 2006, Johannesburg, South Africa; CINVESTAV-IPN, MEXICO

## Abstract

Because of different reported effects of parasitism on the accumulation of metals in fish, it is important to consider parasites while interpreting bioaccumulation data from biomonitoring programmes. Accordingly, the first step is to take parasitism into consideration when simulating metal bioaccumulation in the fish host under laboratory conditions. In the present study, the accumulation of metals in fish-parasite systems was simulated by a one-compartment toxicokinetic model and compared to uninfected conspecifics. As such, metal accumulation in fish was assumed to result from a balance of different uptake and loss processes depending on the infection status. The uptake by parasites was considered an efflux from the fish host, similar to elimination. Physiological rate constants for the uninfected fish were parameterised based on the covalent index and the species weight while the parameterisation for the infected fish was carried out based on the reported effects of parasites on the uptake kinetics of the fish host. The model was then validated for the system of the chub *Squalius cephalus* and the acanthocephalan *Pomphorhynchus tereticollis* following 36-day exposure to waterborne Pb. The dissolved concentration of Pb in the exposure tank water fluctuated during the exposure, ranging from 40 to 120 μg/L. Generally, the present study shows that the one-compartment model can be an effective method for simulating the accumulation of metals in fish, taking into account effects of parasitism. In particular, the predicted concentrations of Cu, Fe, Zn, and Pb in the uninfected chub as well as in the infected chub and the acanthocephalans were within one order of magnitude of the measurements. The variation in the absorption efficiency and the elimination rate constant of the uninfected chub resulted in variations of about one order of magnitude in the predicted concentrations of Pb. Inclusion of further assumptions for simulating metal accumulation in the infected chub led to variations of around two orders of magnitude in the predictions. Therefore, further research is required to reduce uncertainty while characterising and parameterising the model for infected fish.

## Introduction

Fish have widely been used as bioindicators for metal pollution in aquatic systems [1,2,3,4,5]. Moreover, simulation of metal accumulation in fish can provide input required for estimating the risks to fish consumers, especially humans, of exposure to metals via the food chain. Bioaccumulation of chemicals in aquatic species has been well simulated with biodynamic models [6,7,8,9,10,11]. Reasonable estimates of metal bioaccumulation could be obtained under field conditions when organisms are exposed to metal mixtures [8]. Available models for simulating metal accumulation in fish have usually been developed assuming the constancy of the exposure concentration, which does not represent the real conditions either in the laboratory or in the nature [12,13,14].

The number of parameters required as input to a one-compartment model is usually smaller than that for a PBPK (physiologically based pharmacokinetic) model [15,16]. The PBPK model might provide a more mechanistic understanding of metal toxicity and be reasonably extrapolated across different conditions. However, these advantages might be outweighed by the requirement for more inputs than the one-compartment model [17]. Some of the required inputs for the PBPK model are not easily accessible or not available at all [15,16,17]. Consequently, different assumptions are required to parameterise the missing factors, which can lead to uncertainties in the model predictions.

Parasites are able to influence the accumulation of metals in the fish host [18,19,20]. Furthermore, parasites such as the palaeacanthocephalan *Pomphorhynchus laevis* in the fish intestine have been suggested as a potential indicator of metal pollution [21]. The distribution of metals in fish-parasite systems is commonly expressed by a simple ratio between metal concentrations in the parasite and in the fish host tissues [22,23,24,25,26,27]. The exclusion of physiological characteristics of organisms as well as physicochemical properties of the environment in this method limits the potential for extrapolation across species or conditions.

In the present study, we aimed at developing a biodynamic model for simulating metal accumulation in a fish-parasite system following aqueous exposure. The aqueous exposure was selected because of the well-simulated mechanism via which Pb is accumulated in the acanthocephalan following exposure of the chub host to waterborne Pb [18,28]. This knowledge facilitates characterising the model simulating Pb accumulation in the fish-parasite system. Kinetic parameters in the model were related to fish size, as the body size is one of the decisive factors determining metal accumulation in fish [29,30,31,32,33,34]. Such a mechanistic model can be applied to different conditions without the need for calibration for each metal-species combination [8]. The model was developed based on data from previous studies for different parasite and fish species and then validated using an independent data set generated during our laboratory experiments on the system of the chub *Squalius cephalus* and the acanthocephalan *Pomphorhynchus tereticollis*. Due to the lack of data regarding infected fish, some assumptions were required in order to develop and calibrate the biodynamic model. Uncertainties related to these assumptions were also assessed in the present study.

## Methods

### Exposure experiments & biochemical and statistical analyses

The animal experiment (Reference number is 84–02.04.2013.A389) was approved according to European regulations by the Landesamt für Natur-, Umwelt- und Verbraucherschutz (LANUV). Chub fingerlings were obtained from the aquaculture facilities of the Research Institute for Nature and Forest (INBO), Belgium. Fish were kept in 500-L tanks with dechlorinated tap water and fed daily with commercial pellets. During the maintenance, the water was changed twice weekly. The experiments were carried out when the fish reached the weight of 10 (± 3) g. The fish were divided into four groups: uninfected control (G1), infected control (G2), uninfected Pb-exposed (G3), and infected Pb-exposed (G4, see Table 1). Amphipods naturally infected with cystacanths of the acanthocephalan *P*. *tereticollis* were collected from the river Wupper in Germany. Prior to the infection, the cystacanths were dissected from the amphipods using scissors and forceps and transferred to a 0.9% saline solution. Subsequently, the chub in groups G2 and G4 were infected with ten cystacanths each, using a 2-ml syringe fitted with a 12-cm length of 1-mm-diameter plastic tubing into the digestive tract of the fish [21].

**Table 1:** The number and wet weight (average ± SD) of the chub *Leuciscus cephalus* and the acanthocephalan *Pomphorynchus tereticolis* in the group investigated.

Parameters	Control groups			Pb-exposed groups		
	Uninfected	Infected		Uninfected	Infected	
	*Squalius cephalus*	*Squalius cephalus*	*Pomphorhynchus tereticollis*	*Squalius cephalus*	*Squalius cephalus*	*Pomphorhynchus tereticollis*
Number	7	7	5.29 (± 2.21)	7	7	5.71 (± 3.77)
Weight*	7.60 (± 2.41)	10.76 (± 2.83)	1.62 (± 1.11)	9.86 (± 3.86)	10.74 (± 1.64)	1.87 (± 1.13)

*Units of the chub and acanthocephalan weight: g and mg, respectively.

Five weeks after infection, the exposure experiment was conducted. Fish (uninfected or infected) were placed into 20-L tanks filled with 10L of tap water without (control groups) or with (Pb-exposed groups) addition of Pb as nitrate salt. Fish were maintained at room temperature of 20 ± 3°C whereas a light cycle with a ratio of 16:8 (light: dark) was simulated. Water was completely changed once in three days. Water samples were taken before and after changing the water with a 0.45 μm filter for determining dissolved concentrations of Pb and metals that are available in tap water, e.g. Fe, Cu, and Zn (S1 Fig). One day after water renewal, the chub were fed with commercial fish pellets. After 36 days of exposure, the chub were killed by cervical dislocation and the acanthocephalans were removed from the hosts. The number of the acanthocephalans in each chub individual was counted while the acanthocephalans were weighed (Table 1). All acanthocephalans found per fish were pooled and represented one parasite tissue sample for metal analysis.

The fish were homogenised using a tissue homogeniser (Ultra-Turrax T25, IKA-Labortechnik, Staufen, Germany). Subsequently, about 70–100 mg of the samples (wet weight) was weighed in 30 ml Teflon tubes (Xpress vessels: CEM, Germany) and digested in the microwave (MARS 6; CEM, Germany) with 4 mL of HNO_3_ 65% at 180°C. From 6 to 10 blank samples were included in each batch of digestion. Metal concentrations were obtained using inductively coupled plasma mass spectrometry (ICP-MS, Perkin Elmer Elan 6000). The validity of the procedure was checked by using three reference materials, namely IAEA-407 (Fish Homogenate Reference Material, International Atomic Energy, Monaco), DORM-2 (Dogfish Muscle Certified Reference Material, National Research Council, Canada), and DOLT-3 (Dogfish Liver Certified Reference Material, National Research Council, Canada). The recovery rates of Fe, Cu, Zn, and Pb in these reference materials (ranging from 80% to 110%) are given in S1 Table.

As mentioned above, the partitioning of metals in fish-parasite systems is usually expressed by a bioconcentration factor, i.e. the ratio of the metal concentration in the parasites to the concentration in the host or in the host organ. To facilitate comparing the pattern of metal partitioning in the system of the chub and the acanthocephalan with that in other fish-parasite systems, the average and the standard deviation of the bioconcentration factor were calculated in the present study by using R software.

### Model development for uninfected fish

#### Model characterisation

The accumulation of metals in fish occurs as a balance of influxes and effluxes. Following aqueous exposure, uptake via gills has been demonstrated as the major contributor to the accumulation in fish for a number of metals [18,35,36]. The uptake of aqueous metals can be represented by the uptake rate constant *k*_u_ (L/g/d). Metals can be eliminated via gills, kidney, and intestine, and the contribution of these pathways varies among metals and the exposure source [15,16,37]. In our one-compartment model, the elimination was represented by one single rate constant *k*_e_ (1/d). In addition, metal concentrations in fish can be affected by the growth dilution and this factor was also considered in the model. The changing metal concentration in fish can therefore be described by the following equation:

$$\frac{{\text{dC}}_{t}}{\text{dt}}=k_{\text{u}}\times {\text{Cw}}_{\text{i}}-\left(k_{\text{e}}+\text{g}\right)\times {\text{C}}_{t}$$ (1)

where C_*t*_ (μg/g ww) is the metal concentration in the fish based on wet weight (ww); *k*_u_ (L/g/d) is the dissolved uptake rate constant; Cw_i_ (μg/L) is the metal concentration in the exposure solution; *k*_e_ (1/d) is the elimination rate constant, excluding a distinction between different elimination pathways; and g (1/d) is the mass-based growth rate constant.

The accumulation of metals in fish exposed to varying concentrations was modelled by dividing the exposure duration into *n* intervals corresponding to each time of renewal (*T*_*j*_) and assuming that metal concentrations during the intervals were constant. This method has been applied in previous studies on modelling the accumulation of metals from water at varying exposure concentrations [38,39]. The metal concentration in the whole fish at time *t* between the renewal *T*_*j*_ and *T*_*j+1*_ can be calculated according to the following equation, similar to that developed by Adam et al. [38]:

$${\text{C}}_{t}={\text{C}}_{0}\times e^{-\left(k_{\text{e}}+\text{g}\right)\times \left(t-T_{0}\right)}+\frac{k_{\text{u}}}{k_{\text{e}}+\text{g}}\times \left(\sum {\text{Cw}}_{j}\times \left(e^{-\left(k_{\text{e}}+g\right)\times \left(t-T_{j}\right)}-e^{-\left(k_{\text{e}}+\text{g}\right)\times \left(t-T_{j-1}\right)}\right)+{\text{Cw}}_{j+1}\times \left(1-e^{-\left(k_{\text{e}}+\text{g}\right)\times \left(t-T_{j}\right)}\right)\right)$$ (2)

where C_0_ (μg/g ww) is the initial metal concentration in the uninfected fish; *k*_e_ (1/d) is the elimination rate constant; g (1/d) is the growth rate constant; and ${\text{C}}_{{\text{W}}_{j}}$ (μg/L) is the metal concentration in the tank water during the interval *j*.

A detailed description of the mathematical derivation for determining metal concentrations in the whole fish exposed to fluctuating exposure concentrations is given in S1 File.

### Model parameterisation

**Data collection and processing:** Only data generated from experiments similar to the experiments in the present study were used to parameterise the model. Therefore, only data fulfilling the following criteria was considered: 1) Calculation was carried out based on dissolved metal concentrations; 2) Experimental settings were similar to our experiments, i.e. static experiments and regular renewal of tank water; 3) Fish were exposed to metals in the dissolved phase only; and 4) Exposure durations were longer than one week.

**Model parameterisation**: Both metal toxicokinetics (represented by the dissolved uptake rate constant and the elimination rate constant) and the growth rate constant have been demonstrated to be explained by the fish size [40].

*Dissolved uptake rate constant*: The dissolved uptake rate constant varies between metals and species. The uptake of metals by fish is highly correlated to the affinity of metal for binding sites in the fish [41]. This has been translated into a relationship between the absorption efficiency *p* (%) and chemical-specific properties of metals [8,42,43]. Moreover, the intra- and inter-species variations could be taken into account by including size dependence in simulating the ventilation rate [8,42]. In other words, the dissolved uptake rate constant can be expressed as:

$$k_{\text{u}}=p\times \text{VR}$$ (3)

where *p* (%) is the metal-specific absorption efficiency; and VR (L/g/d) is the size-related ventilation rate.

The ventilation rate is limited by the metabolism of enzymes in the liver and this limitation can be described by a Michaelis-Menten equation [44]:

$$\text{VR}=\frac{{\text{Q}}_{\text{Liver}}\times {\text{VR}}_{\text{max}}}{{\text{Q}}_{\text{Liver}}+{\text{VR}}_{\text{max}}}$$ (4)

where VR (L/g/d) is the ventilation rate used as input to the model; Q_Liver_ (99.14· 10^−3^ L/g/d) is the liver blood flow, calculated from the arterial blood flow to liver compartment determined by Nichols et al. [44]; and VR_max_ (L/g/d) is the maximal ventilation rate derived from previous studies as described below.

In the study of Arnot and Gobas [45], exposure conditions such as the dissolved oxygen content were considered determinants of the ventilation rate. However, these conditions are usually lacking in the studies on metal uptake in fish. Therefore, the equation developed by Arnot and Gobas [45] was not used to simulate the ventilation rate in the present study. Instead, the method developed by Nichols et al. [46,47] was used. Nichols et al. [46,47] assumed that the ventilation rate scales to species weight with a coefficient of -1/4, similar to the approach applied in a number of other studies (e.g. Le et al. [8]):

VRmax=254.4×10−3×(10−3×W)−14 (5)

where W (g) is the wet weight of the whole fish.

The metal-specific absorption efficiency was first calculated by using the ventilation rate determined by the above approach and the collected data on the dissolved uptake rate (S2 Table). Several chemical properties of metals have been reported to be related to the uptake, bioaccumulation, and toxicity of metals [8,42,48,49,50]. These chemical properties include the ionic radius, the electronegativity, the covalent index, the softness index, the first hydrolysis constant, and the ionic index. Our assessment showed that among these properties, the covalent index ($\chi_{\text{m}}^{2}\text{r}$) is the best predictor of the absorption efficiency (S3 Table). Consequently, the derived relationship between the absorption efficiency and the covalent index was used to obtain the input for the absorption efficiency to the model:

$$\text{log}\left(\frac{p}{1-p}\right)=0.18\times \chi_{\text{m}}^{2}\text{r}-2.31$$ (6)

*Elimination rate*: Elimination rates vary depending on exposure routes [6,51,52,53,54]. The differences in elimination between the exposure routes are related to the internal distribution of metals, which is dependent on the routes [53]. Therefore, only elimination rates measured following dissolved exposure were used in the present study. In some studies, biexponential elimination was used for simulating the varying concentration of metals in organisms during the depuration phase [55]. As such, slow- and fast compartments have been discriminated in simulating the elimination of metals in fish [38,39,56,57]. However, experiments do not usually last long enough to consider such distinction in the simulation [58]. The elimination rates determined by considering these different depuration phases were therefore not used for model parameterisation in the present study. Instead, the elimination rate was recalculated from the raw data obtained in depuration experiments. The collected data on elimination rates used for model parameterisation are given in S4 Table.

Elimination rates depend on water temperature [58,59,60]. However, limited data hinder developing a quantitative relationship between elimination rates and water temperature [56]. Therefore, the dependence of elimination rates on water temperature was excluded in our model, similar to the approaches applied previously [8,42]. As such, temperature was not considered in extrapolating the elimination rate determined in previous studies to the present study. Similarly, the concentration of metals in water was excluded in simulating the elimination rate, following previously published findings that metal elimination following aqueous exposure is independent of the exposure level [61].

No significant differences in elimination rates among fish species with the same range of weight have been reported [54]. This is consistent with the conclusion by Norey et al. [62] that elimination does not account for the inter-species variations in metal accumulation. Furthermore, Dutton and Fisher [54] showed that elimination among different species could be explained by metabolic processes. Based on these findings we assumed that the inter-species variations in elimination rates can be explained by the fish size. Negative relationships between the elimination rate and the fish size have been reported in a number of previous studies [40,56,57,58,63,64]. It has usually been assumed that the variations in the elimination rate with varying fish size can be explained by an allometric equation of metabolic processes [8,42,65]. However, empirical allometric equations developed are very scarce. Until now, such equations were developed for mercury only, with various allometric exponents from -0.22 to -0.58 [56]. In the study of Trudel and Rasmussen [56], data used for establishing a relation between the elimination rate and the fish size have been generated in experimental conditions that are considerably different from those in the present study. Therefore, the relationship derived by these authors was not used in our modelling. Instead, the elimination rate was modelled by the following equation:

ke=ke,0×W−14 (7)

where *k*_e_ (1/d) is the elimination rate constant for the fish with the weight W (g); and *k*_e,0_ (1/d) is the weight-corrected elimination rate constant.

Similar to the parameterisation of the absorption efficiency as described above, the weight-corrected elimination rate derived from the collected data (S4 Table) was related to chemical properties of metals. The covalent index ($\chi_{\text{m}}^{2}\text{r}$) was chosen in the parameterisation of the elimination rate because of its highest capacity for explaining the variations in the elimination between metals, compared to other chemical properties (S5 Table):

$$\text{log}k_{\text{e},0}=0.25\times \chi_{\text{m}}^{2}\text{r}-1.78$$ (8)

*Growth rate*: The growth of the juvenile chub *S*. *cephalus* has been investigated in a number of studies. While the growth of larval *S*. *cephalus* under controlled laboratory conditions has been studied [66,67,68], the growth of the adult has been examined in field studies only [69,70,71,72,73,74,75]. The field studies show the dependence of the growth of adult chub on environmental conditions. Therefore, the relative growth rate measured in these studies was not used for model parameterisation in the present study. Instead, the relative growth rate in weight was determined from a growth experiment at the same conditions as in our exposure experiments for 36 days.

The relative growth rate in weight can be described by the following equation:

$$\text{g}=\frac{{\text{W}}_{t}-{\text{W}}_{t-1}}{{\text{W}}_{t-1}}$$ (9)

where g (1/d) is the relative growth rate constant in weight; W_*t*_ (g) is the weight of the whole fish after *t* days; and W_*t*-1_ (g) is the weight of the whole fish after *t*-1 days.

The weight of the whole fish could therefore be calculated from the relative growth rate in weight as:

$${\text{W}}_{t}={\text{W}}_{0}\times {\left(1+\text{g}\right)}^{t}$$ (10)

where W_0_ (g) is the initial weight of the fish.

The relative growth rate constant in weight was subsequently calculated by optimising the similarities between the weight of the whole fish predicted from Eq 10 and the weight measured in the experiment (Table A in S2 file). This method is based on the assumption that the growth of chub was not limited or during the short exposure period, the weight of chub did not reach the maximum. Following this method, the growth rate constant of 0.015 (1/d) was determined by using Sigma Plot.

### Model development for parasite-fish systems

#### Model characterisation

The metal uptake by parasites was considered another efflux of metal accumulation in the host (Eq 11):

$$\frac{{\text{dC}}_{t}}{\text{dt}}=k_{\text{u}}\times {\text{Cw}}_{\text{i}}-\left(k_{\text{e}}+\text{g}+k_{\text{p}}\right)\times {\text{C}}_{t}$$ (11)

where C_*t*_ (μg/g ww) is the metal concentration in the whole fish; *k*_u_ (L/g/d) is the rate constant of metal uptake via the dissolved phase; Cw_i_ (μg/L) is the dissolved concentration of the metal in the exposure solution; *k*_e_ (1/d) is the elimination rate constant; g (1/d) is the growth rate constant; and *k*_p_ (1/d) is the uptake rate constant by the parasites.

In addition, metal concentrations in parasites can be simulated by another mass balance equation:

$$\frac{{\text{dC}}_{\text{p}}}{\text{dt}}=k_{\text{p}}\times {\text{C}}_{t}\times \frac{\text{W}}{{\text{W}}_{\text{p}}}-n_{\text{p}}\times {\text{g}}_{\text{p}}\times {\text{C}}_{\text{p}}$$ (12)

where C_p_ (μg/g ww) is the metal concentration in parasites; W (g) is the whole fish weight; W_p_ (g) is the weight of the parasites; *n*_p_ is the number of parasite individuals inhabiting the fish host; and *g*_p_ (1/d) is the growth rate constant of the parasites. This equation can be re-written in a similar way for the accumulation of metals in fish from the exposure solution:

$$\frac{{\text{dC}}_{\text{p}}}{\text{dt}}=k_{\text{p}}\times {\text{Cf}}_{i}-n_{\text{p}}\times {\text{g}}_{\text{p}}\times {\text{C}}_{\text{p}}$$ (13)

where Cf_i_ (μg/kg ww) represents the source of metals from the fish:

$${\text{Cf}}_{i}={\text{C}}_{t}\times \frac{\text{W}}{{\text{W}}_{\text{p}}}$$ (14)

where C_*t*_ (μg/kg ww) is the metal concentration in fish; W (g) is the whole fish weight; and W_p_ (g) is the weight of the parasites inhabiting the fish.

Similar to the uptake by fish from the solution, we assumed limited variations in the metal source from the host for the parasites during the 3-day intervals. Based on this assumption, metal concentrations in the parasites (C_p_) and infected fish (C_t_) can be elaborated similarly to the approach for metal accumulation in uninfected fish as described above:

$${\text{C}}_{\text{p}}={\text{C}}_{0}^{*}\times e^{-n_{\text{p}}\times {\text{g}}_{\text{p}}\times \left(t-T_{0}\right)}+\frac{k_{\text{p}}}{n_{\text{p}}\times {\text{g}}_{\text{P}}}\times \left(\sum {\text{Cf}}_{j}\times \left(e^{-n_{\text{p}}\times {\text{g}}_{\text{p}}\times \left(t-T_{j}\right)}-e^{-n_{\text{p}}\times {\text{g}}_{\text{p}}\times \left(t-T_{j-1}\right)}\right)+{\text{Cf}}_{j+1}\times \left(1-e^{-n_{\text{p}\times }{\text{g}}_{\text{p}}\times \left(t-T_{j}\right)}\right)\right)$$ (15)

where ${\text{C}}_{0}^{*}$ (μg/g ww) is the initial concentration of metals in the parasites; *n*_p_ is the number of the parasites inhabited in the fish host; g_p_ (1/d) is the growth rate constant of the parasites; *k*_p_ (1/d) is the uptake rate constant by the parasites; and Cf_*j*_ (μg/g) is the metal source from the fish;

$${\text{C}}_{t}={\text{C}}_{0}\times e^{-\left(k_{\text{e}}+\text{g}+k_{\text{p}}\right)\times \left(t-T_{0}\right)}+\frac{k_{\text{u}}}{k_{\text{e}}+\text{g}+k_{\text{p}}}\times \left(\sum {\text{Cw}}_{j}\times \left(e^{-\left(k_{\text{e}}+\text{g}+k_{\text{p}}\right)\times \left(t-T_{j}\right)}-e^{-\left(k_{\text{e}}+\text{g}+k_{\text{p}}\right)\times \left(t-T_{j-1}\right)}\right)+{\text{Cw}}_{j+1}\times \left(1-e^{-\left(k_{\text{e}}+\text{g}+k_{\text{p}}\right)\times \left(t-T_{j}\right)}\right)\right)$$ (16)

where C_0_ (μg/g ww) is the initial concentration of metals in the infected fish; *k*_e_ (1/d) is the elimination rate constant of metals in the infected fish; g (1/d) is the growth rate constant of the fish; *k*_p_ (1/d) is the uptake rate constant by the parasites; and *k*_u_ (L/g/d) is the rate constant of metal uptake by the infected fish via the dissolved phase.

### Model parameterisation

**Absorption efficiency and elimination rate:** According to Oyoo-Okoth et al. [76], infestation of fish with parasites affected the uptake and elimination of metals by the fish host. In the present study, effects of parasitism on the rate of metal uptake by the host were accounted for by including the influence on the absorption efficiency, assuming that parasitism does not have effects on the ventilation rate. No information on the uptake and elimination rates of infected fish is available for Pb. Therefore, two scenarios (Table 2) were considered based on the findings by Oyoo-Okoth et al. [76] on the uptake kinetics of Cd and Co in the system of the cyprinid fish *Rastrineobola argentea* and the tapeworm *Ligula intestinalis*. In the first scenario, the uptake kinetics of Pb in the host was assumed to be affected by parasites in the same way as the uptake of Cd: the uptake rate by the infected fish is two times higher than the rate for the uninfected fish and the elimination rate in the infected fish is 1.7 times lower than the rate for the uninfected. In the second scenario, parasitism was assumed to have the same effects on the uptake kinetics of Pb and Co: Pb is taken up by infected fish at a rate two times lower than by the uninfected while being eliminated from infected fish at 1.7 times higher than from the uninfected.

**Table 2:** Scenarios examined for assessing sensitivity of the modelled metal concentration in the parasite-fish system.

Parameters		Standard scenarios		Absorption-efficiency-scenarios				Elimination-rate-scenarios			
		S1	S2	A1	A2	B1	B2	C1	C2	D1	D2
Absorption efficiency	Uninfected chub	Default	Default	2 times lower than Default	2 times lower than Default	2 times higher than Default	2 times higher than Default	Default	Default	Default	Default
	Infected chub	Act like Cd	Act like Co	Act like Cd	Act like Co	Act like Cd	Act like Co	Act like Cd	Act like Co	Act like Cd	Act like Co
Elimination rate	Uninfected chub	Default	Default	Default	Default	Default	Default	2 times lower than Default	2 times lower than Default	2 times higher than Default	2 times higher than Default
	Infected chub	Act like Cd	Act like Co	Act like Cd	Act like Co	Act like Cd	Act like Co	Act like Cd	Act like Co	Act like Cd	Act like Co

**Relative growth rate of fish:** The relative growth rate in weight of fish in a short investigation period was assumed not to be affected by parasites. This assumption is based on the negligible differences in the weight of uninfected and infected chub observed in previous studies [20,77].

**Relative growth rate of parasites:** The relative growth rate of individual parasites in weight was parameterised by the same approach as applied for fish, as described above. The growth rate constant of 0.021 (1/d) was obtained by using the data in the study of Sures et al. [20] for *P*. *laevis* with Sigma Plot.

**Uptake rate of parasites:** The uptake rate constant of parasites (1/d) was determined from Eq 16 by using data in the study of Sures et al. [20]. As such, the time-dependent metal concentration in the whole fish is required for the parameterisation of the uptake rate of the parasites. However, this information was lacking in the study of Sures et al. [20]. In the parameterisation of the uptake rate of parasites, metal concentrations in the whole fish were estimated from the concentration in muscle, based on significant relationships between metal concentrations in the whole fish and in muscle as reported in previous studies [78,79] and described in the following part. Linear relationships have been found between metal accumulation in the whole fish and in muscle based on the data previously published in other studies [79,80]. For example, concentrations of Hg in herring and perch muscle were strongly and significantly related to the concentrations in the whole fish according to a linear relationship [79]. This is consistent with the results based on the data generated by Sures et al. [20] as presented in S2 Fig. Moreover, such a strong and significant relationship between the concentration in muscle and the concentration in the whole fish held for a variety of metals, i.e. Fe, Mn, Zn, Cu, Pb, Ni, and Cd (Honda et al. [80]; S3 Fig). Therefore, in the parameterisation of the uptake rate by parasites, the Pb concentration in the whole fish was estimated from the concentration in muscle and used in further steps as presented in the detailed description given in S2 File. Such parameterisation resulted in a value of the Pb uptake by parasites of 1.36· 10^−3^ (1/d). The lack of data on the time-dependent accumulation of Fe, Cu, and Zn in fish-parasite systems prevents us from parameterising the uptake rate constant of parasites for other metals. Therefore, we applied the uptake rate constant determined above for Pb to other metals as well.

### Model validation for the chub-acanthocephalan system

In generating model predictions, the concentration of metals in the uninfected control chub and in the acanthocephalans inhabiting the control chub was considered the initial concentration of the metals in the chub and in the acanthocephalans, respectively. The performance of the developed model was evaluated by comparing the analysed metal concentration in the uninfected chub, the infected chub, and the acanthocephalans with the corresponding predicted concentration according to the developed model by using different means of statistical parameters. The capacity of the model in explaining the variations in the metal concentration in the chub or in the acanthocephalans was expressed by the value of *r*^2^ and *p* [81]. In addition, the deviation between the measured and the predicted concentrations was represented by the values of mean absolute error (MAE) and root mean square error (RMSE) [81]. The validation for Fe, Cu, and Zn at the background concentrations of the tap water was carried out assuming that at those low concentrations, the accumulation of the metals in the chub was not affected by the acanthocephalans.

### Uncertainty and sensitivity analyses

In the present study, the absorption efficiency and the elimination rate constant of the uninfected and infected fish were parameterised based on data for various metals as well as for different fish and parasite species. Therefore, they are potential sources of uncertainty in applying the developed model to specific systems, for example, to predict the accumulation of Cu, Zn, Fe, and Pb in the chub-acanthocephalan system in our validation. The sensitivity of the estimates of metal concentrations in the chub-acanthocephalan system was assessed by examining the variations in the predicted Pb concentration in the uninfected chub as well as in the infected chub and in the acanthocephalans with varying the absorption efficiency and the elimination rate constant within a factor of 2 (Table 2).

## Results

### Metal accumulation in chub and acanthocephalans

**Fig 1:**
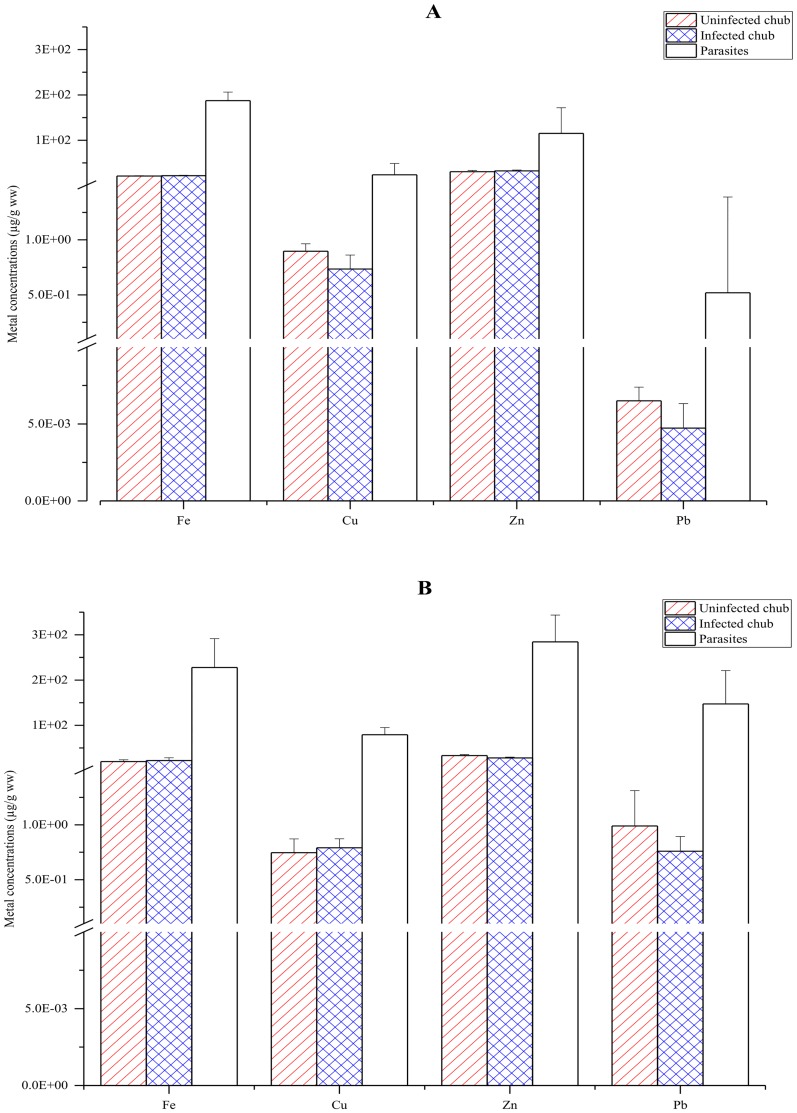
Metal concentrations in the uninfected chub, the infected chub, and the acanthocephalans based on wet weight (ww) when the chub is not exposed to Pb (A) and when the chub is exposed to Pb (B). The error bars represent the standard deviation. The slashes on the y-axis represent the breaks.

For all investigated metals (Fe, Cu, Zn, and Pb), there were no significant differences in the accumulation level in the uninfected chub compared to that in the infected chub as shown by the overlapping ranges (average ± standard deviation) of the measured metal concentrations in the uninfected and infected chub (Fig 1). Moreover, the concentrations of these metals in both the uninfected and infected chub were significantly lower than those in the acanthocephalans when the ranges (average ± standard deviation) of the measured metal concentrations in the chub and in the acanthocephalans did not overlap each other (Fig 1).

The concentrations of Fe, Cu, and Zn in the acanthocephalans and in the chub host when the host was not exposed to Pb were similar to those when the host was exposed to Pb (Fig 1). Similarly, the concentrations of these metals in the chub of the uninfected control group were not significantly different from those in the chub of the uninfected Pb-exposed group (Fig 1). This indicates that at the background exposure concentration in the tap water, the accumulation of essential metals like Cu, Fe, and Zn in the chub and the acanthocephalans was not affected by the addition of Pb to the exposure solution. As a result, the Pb exposure reduced the differences between the concentrations of essential metals and of Pb in the chub as well as in the acanthocephalans (Fig 1).

The partitioning in the chub-acanthocephalan system differed between essential metals (Fe, Cu, and Zn) and the non-essential metal (Pb) (Fig 1). In particular, when the chub host was not exposed to Pb, the deviations between the concentrations in the acanthocephalans and in the host was substantially smaller for Fe, Cu, and Zn (around one order of magnitude) than for Pb (more than two orders of magnitude) (Fig 1A). Without the Pb exposure, Fe and Zn were accumulated by the acanthocephalans and the chub host at the highest level, being significantly higher than the accumulation level for Cu (Fig 1A). In the system not exposed to Pb, the concentrations of the essential metals in the chub host were up to four orders of magnitude higher than the corresponding concentrations of Pb, while in the acanthocephalans, the difference between the concentrations of the essential metals and of Pb ranged from one to two orders of magnitude (Fig 1A). Following the Pb exposure, the concentration of Cu in the uninfected chub did not significantly deviate from that of Pb (Fig 1B). A similar observation was found for the concentration of these two metals in the acanthocephalans as well as in the chub host (Fig 1B).

When the chub were not exposed to Pb, the partitioning between the acanthocephalans and the chub host, as expressed by the bioconcentration factor was most unequal for Pb, compared to Fe, Cu, and Zn (Table 3). However, the difference was not statistically significant as shown by the overlapping range (average ± standard deviation) of the calculated bioconcentration factors, attributed to the large variation in the bioconcentration factor for Pb (Table 3). The variations in the bioconcentration factors were larger when the chub host was exposed to Pb than when the chub host was not exposed to Pb (Table 3). When the chub host was exposed to Pb, Pb and Cu had the highest bioconcentration factor values, significantly higher than those for Fe and Zn (Table 3). The exposure of the chub host to Pb increased the partitioning of Pb to the acanthocephalans, but not significantly, while there were significant increases in the partitioning of Cu and Zn to the acanthocephalans (Table 3).

**Table 3:** Bioconcentration factors (i.e. the ratio between the metal concentration in parasites and the concentration in the whole fish or in the muscle) for different metals found in the present study or reported in previous studies.

Fish-parasite system		Experimental set-up	Fe	Cu	Zn	Pb	Other metals	References
Fish	Parasite							
*Squalius cephalus*	*Pomphorhynchus tereticollis*	Lab exposure experiment/Water	8.58 (± 0.90)	32.00 (± 34.23)	3.58 (± 1.74)	140 (± 237)		Present study^a^
			9.07 (± 0.50)	102.96 (± 35.25)	10.16 (± 1.24)	178 (± 57)		Present study^b^
*Squalius cephalus*	*Pomphorhynchus laevis*	Field sampling					400–2700 (Cd, Pb)	Sures and Siddall [18]
*Barbus barbus*	*Pomphorhynchus laevis*	Field sampling					0.2–600^d^	Thielen et al. [24]
*Perca fluviatilis*	*Acanthocephalus lucii*	Field sampling		67.7	10.1	52.1		Brazova et al. [26]^c^
	*Proteocephalus perca*			19.8	8.7	21.3		
*Perca fluviatilis*	*Pomphorhynchus laevis*	Field sampling	3.3. (± 2.8)	26.0 (± 26.5)	10.3 (± 4.7)	337 (± 401)		Nachev et al. [82]^c^
	*Eustrongylides sp*		14.3 (± 7.7)	122.9 (± 71.0)	11.6 (± 6.1)	9.4 (± 12.5)		
*Oncorhynchus mykiss*	*Raphidascaris acus*	Laboratory exposure experiment/Dietary					0.09 (Se)	Hursky and Pietrock [85]
*Nemipterus peronii*	*Hysterothalycium reliquens*	Field sampling	27.02 (± 9.8)	60.1 (± 5)	33.3 (± 9)	6.7 (± 4.3)	185 (± 0.06) (Cd)	Mazhar et al. [87]^c^
	*Paraphilometroides nemipteri*		25.4 (± 5.4)	52.3 (± 2)	24.2 (± 7)	11 (± 1.9)	292 (± 0.06) (Cd)	
*Oreochromis niloticus*	*Acanthogyrus* sp.	Field sampling				147		Paller et al. [97]^c^
*Perca fluviatilis*	*Acanthocephalus lucii*	Field sampling	11.45	240	20.79			Sures [109]
*Barbus barbus*	*Pomphorhynchus laevis*	Field sampling					26–407 (Cd, Pb, Zn)	Schludermann et al. [110]
*Notothenia coriiceps*	*Aspersentis megarhynchus*	Field sampling	7	81		325		Sures and Reimann [111]^c^
*Perca fluviatilis*	*Acanthocephalus lucii*	Field sampling	11	250	33			Sures et al. [112]
*Perca fluviatilis*	*Acanthocephalus lucii*	Field sampling		24.4	4.7			Jankovska et al. [113]^c^

^a^Determined in control experiment, i.e. the chub is exposed to metals at the background concentrations of the tap water;

^b^Determined in Pb exposure experiment;

^c^Metal concentrations in the muscle of the host were used to represent the concentration in the whole host because of the dominant contribution of this organ to the total weight of the host;

^d^Multiple metals (As, Al, Ag, Ba, Bi, Ca, Cd, Co, Cr, Cu, Fe, Ga, Mg, Mn, Ni, Pb, Sb, Sn, Sr, Tl, V, Zn)

### Validation results for the chub-acanthocephalan system

**Fig 2:**
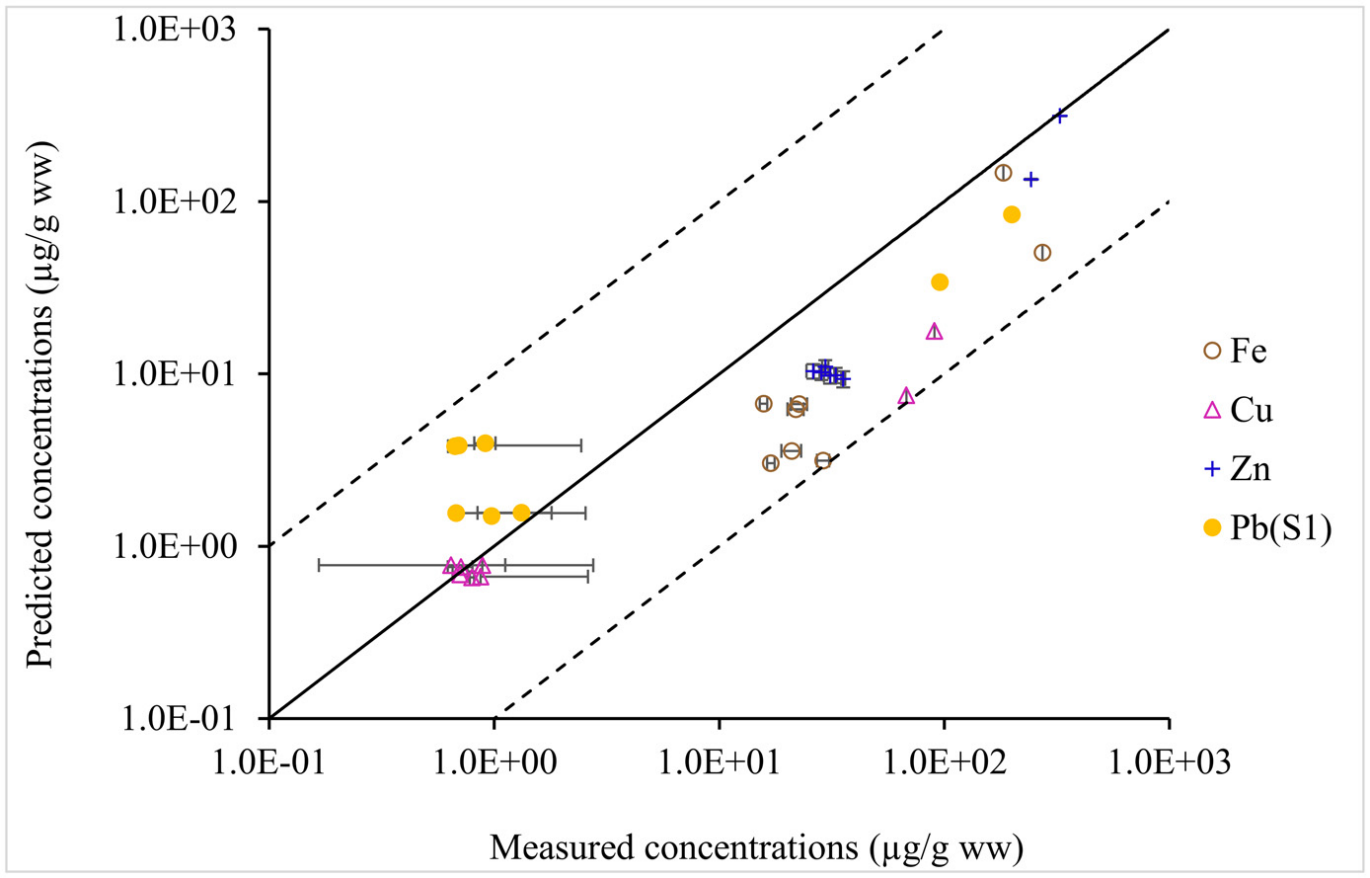
Comparison of the predicted and the measured concentrations of Fe, Cu, Zn, and Pb in the chub-acanthocephalan system. The horizontal error bars represent the standard deviation in the measurement of metal concentrations. The solid line represents the 1:1 ratio (i.e. the measured concentration equals the predicted concentration). The dashed lines represent the deviation of one order of magnitude from the 1:1 ratio. The model predictions for Pb accumulation in the chub-acanthocephalan system obtained in scenario S1 with the default absorption efficiency and elimination rate were given to provide an illustration on the model performance.

The concentrations of essential metals (i.e. Fe, Cu, and Zn) in the chub-acanthocephalan system were slightly underestimated (Fig 2). However, the predicted concentrations of these essential metals accumulated in the uninfected chub as well as in the chub-acanthocephalan system from the tap water at the background concentrations were within one order of magnitude of the analysed concentrations (Fig 2 and Table 4). More than 90% of the variations in Fe and Zn concentrations in the uninfected chub, the infected chub, as well as in the acanthocephalans could be explained by the model (Table 4). At the same time, the model could explain only around 50% of the varying concentrations of Cu accumulated in the chub-acanthocephalan system, related to the larger variations in the measurements (Fig 2).

**Table 4:** Statistical parameters (including the correlation coefficient *r*^2^, *p*, the mean absolute error MAE, and the root mean square error RMSE) describing the relationship between the modelled and measured concentrations of essential metals (Fe, Cu, and Zn) in the uninfected and infected chub and the parasites at the background concentrations of the tap water.

Statistical parameters	Fe	Cu	Zn
*r*^2^	0.50	0.91	0.93
*p*	< 0.05	< 0.0005	< 0.00001
MAE	44.54	16.70	30.58
RMSE	81.02	33.39	42.57

Similar to the predictions for the essential metals, statistically significant relationships were found between the predicted and the analysed concentrations of Pb in the uninfected chub as well as in the chub-acanthocephalan system using the default absorption and elimination rates (Fig 2 and Table 5). More than 96% of the variations in the Pb concentrations in the chub-acanthocephalan system could be explained by the model developed. Moreover, the accumulation of Pb in the uninfected chub could be simulated well by using the absorption efficiency and the elimination rate constant parameterised from the covalent index and species weight by using data generated for other fish-parasite systems. This is shown by the deviations of less than one order of magnitude between the estimates and the measurements of Pb concentrations in the standard scenario S1 (Fig 2). Based on the assumption that parasitism has the same effects on the uptake kinetics of Pb and Cd (scenario S1), concentrations of Pb accumulated in the chub-acanthocephalan system was overestimated, although still within one order of magnitude of the measurements (Table 5). The assumption that the infection affects the uptake kinetics of Pb and Co in the same way (scenario S2) resulted in lower predicted Pb concentrations in the chub host than the assumption in scenario S1.

**Table 5:** Statistical parameters (including the correlation coefficient *r*^2^, *p*, the mean absolute error MAE, and the root mean square error RMSE) describing the relationship between the modelled and measured concentrations of Pb in the uninfected and infected chub and in the parasites in different scenarios.

Statistical parameters	S1	S2	A1	A2	B1	B2	C1	C2	D1	D2
*r*^2^	0.99	0.97	0.99	0.97	0.99	0.97	0.99	0.97	0.99	0.96
*p*	< 0.00001	< 0.00005	< 0.00001	< 0.00005	< 0.00001	< 0.00005	< 0.00001	< 0.00005	< 0.00001	< 0.00005
MAE	23.46	35.10	30.02	36.12	10.74	33.67	20.57	34.21	27.23	35.95
RMSE	46.26	73.72	62.17	75.93	15.40	69.30	38.85	71.36	55.50	75.63

The estimates of Pb accumulation in the chub-acanthocephalan system were more sensitive to the absorption efficiency than to the elimination rate as shown by larger variations in the predicted concentrations among the absorption-efficiency scenarios (A1, A2, B1, B2) compared to the elimination scenarios (C1, C2, D1, D2) (Table 5). Varying the absorption efficiency within a factor of 2 from the default value resulted in variations of less than one order of magnitude in the predicted concentrations of Pb in the uninfected chub, larger than the variations related to the varying elimination. When the absorption efficiency in the uninfected fish varied by a factor of 2, there were almost two-order-of-magnitude variations in the predicted concentration of Pb in the acanthocephalans and in the chub host, larger than the variations with varying elimination rate constants. Moreover, these results indicate additional uncertainties in predicting metal accumulation in the chub-acanthocephalan system, related to the lacking information on the uptake kinetics of the infected fish.

## Discussion

### Metal accumulation in fish-parasite systems

The order of the concentrations of Cu, Zn, and Fe accumulated in the acanthocephalans found in the present study is consistent with the results reported by Nachev et al. [82] and by Javed and Usani [83]. The decrease in the concentrations in the order: Zn > Cu > Pb observed in the perch *Perca fluviatilis* by Brazova et al. [26] agreed with the results obtained in the present study. The high concentration of Fe found in fish might be related to its specific metabolism with no complete excretion, hindering the metal from being eliminated [84]. The high concentration of Fe and Zn in fish may additionally be due to their physiological functions. For example, Fe is included in Fe-heme compounds, e.g. hemoglobin or myoglobin, and bound to proteins like ferritin [22], while Zn is the constituent of a number of enzymes [84]. Generally, the bioconcentration factors found in the present study were in a range reported previously (Table 3). The dominant partitioning of metals to the acanthocephalans over to the chub host found in the present study is in agreement with the trend reported in a number of other studies for other fish-parasite systems (Table 3). This was also seen when the concentration of metals in parasites was compared with the concentration in fish tissues (Table 3). However, Hursky and Pietrock [85] found an opposite observation when the trout was exposed to Se via dietary pathway.

In consistence with previous studies [20,22,23,86], the present study shows that the concentration of toxic metals like Pb in parasites was many folds higher than that in the fish host. Although a higher concentration in parasites compared to that in the fish host was also consistently found for essential metals like Fe and Zn, the partitioning for these metals was more equal than the partitioning for non-essential metals like Pb (Table 3). Moreover, the high bioconcentration factor of Pb (Table 3) as well as the smaller differences between the average concentration of Pb and the concentration of the essential metals in the acanthocephalans compared to the differences in the chub host (Fig 1A) are in line with the findings by Nachev et al. [82] that the acanthocephalans primarily accumulate toxic metals like Pb. In combination with these results, the larger differences between the concentrations in the acanthocephalans and in the chub host for Pb compared to the differences for Fe, Cu, and Zn indicate some mechanisms that enhance the accumulation of non-essential metals like Pb in the acanthocephalans. Mazhar et al. [87] also found the highest bioconcentration factor in the system of the bream *Nemipterus peronii* and the nematode *Paraphilometroides nemipteri* for Cd. Previous studies (e.g. Sures and Siddall [18]; Sures et al. [28]) have suggested that non-essential metals such as Pb are accumulated in bile-complexes by the acanthocephalan. Therefore, the more unequal partitioning of non-essential metals like Pb and Cd in fish-parasite systems than the partitioning for essential elements might be related to the differences in binding of these metals to steroids in bile and/or excretion of the metals to the host intestine. According to Stamp and Jenkins [88], the production of bile acids is induced when the amount of metals such as Cu and Fe exceeds the levels needed. In the present study, the chub were exposed to Cu and Fe at the background concentration of the tap water and at these exposure levels, Cu and Fe accumulated in the fish might be bound to bile acids at lower extent than Pb. This might account for the less unequal partitioning of Fe, Cu, and Zn in the chub-acanthocephalan system. The lower concentration of Se in the nematode *Raphidascaris acus* compared to the concentration in the trout *Oncorhynchus mykiss* as found by Hursky and Pietrock [85] might be related to the limited induction of bile acids due to the low concentration of Se in the investigated trout. In particular, the concentration of Se in the trout investigated in the study of Hursky and Pietrock [85] was lower than the concentration of Se in the muscle of the bream in the study of Mazhar et al. [87] by more than one order of magnitude.

The above discussion shows metal-specific partitioning in fish-parasite systems. In addition, the partitioning of metals in fish-parasite systems depends on a number of other factors, such as characteristics of the parasite, the developmental stage of the parasite, and the parasite’s location in the host. For example, the accumulation of metals in fish-parasite systems varies among parasites. Acanthocephalans and tapeworms inhabiting the intestine of the fish host are usually able to accumulate metals at high levels [89,90]. In agreement with other studies, the present study shows the ability of intestinal parasites to concentrate metals when the fish host is exposed to waterborne metals (Table 3). The mechanism of metal accumulation in the acanthocephalans following exposure of the fish host to waterborne metals has been simulated in previous studies [18]. Metals are mainly taken up via the gills, entering the bloodstream, besides the paracellular diffusion across the epithelial membrane [18,35,36,91,92]. In the blood, metals are bound to the membrane of erythrocytes and transported to various fish organs. Subsequently, metals can form organometallic complexes with steroids in the bile and transported to the intestine [18,86]. These complexes are taken up by the acanthocephalans together with other essential compounds that cannot be synthesised by the acanthocephalans. Compared to intestinal parasites, a lower ability to concentrate metals has been reported for cestode larvae in the body cavity of the intermediate host [93,94]. The determinants of metal partitioning in fish-parasite systems as mentioned above have been demonstrated to be involved in the accumulation of organic compounds as well [95].

The differences between the concentrations of Fe, Cu, Zn, and Pb in the uninfected chub and in the infected chub were consistently insignificant in the present study, similar to the observation by Genc et al. [96]. In particular, Genc et al. [96] demonstrated that the accumulation of Cd, Cr, Cu, Fe, Hg, Mn, Pb and Zn in the European eel *Anguilla anguilla* infected with the nematode *Anguillicola crassus* was not significantly different from that in the uninfected eel. By contrast, infection with parasites has been reported to reduce the concentration of metals accumulated in the fish host in many of previous studies [18,20,85,97]. Sures and Siddall [18] and Sures et al. [20] reported lower concentrations of Pb in the chub infected with the acanthocephalan *P*. *laevis* than in the uninfected chub. In the study of Hursky and Pietrock [85], the infection with the nematode *R*. *acus* reduced metal concentrations in the trout *O*. *mykiss*. Similarly, the concentration of Pb in the tilapia *Oreochromis niloticus* infected with the acanthocephalan *Acanthogyrus* sp. was significantly lower than that in the uninfected fish [97]. The reduction in the concentration of metals accumulated in the fish host related to the infection with parasites as reported in these studies does not indicate positive effects of the infection on the health of the fish host. For instance, the trout exposed to Se and infected with the nematode had higher energetic demands than the trout exposed to Se alone [85]. The mortality of guppies *Poecilia reticulata* infected with the monogenean *Gyrodactylus turnbulli* increased with increasing Zn concentrations while this was not seen for the uninfected guppies [98]. This indicates that parasites might have effects on metal distribution and metabolism in the fish host, besides the effects on the uptake kinetics.

### Biodynamic models for metal accumulation in fish-parasite systems

The potential of our proposed modelling based on body size of fish strengthens the results obtained in previous studies that this biological trait might act as a determinant of the inter- and intra-species variations in metal accumulation [8,42,65,99,100,101]. Results in the present study demonstrate that the biodynamic model in which metal accumulation is assumed to occur as a balance of different effluxes and influxes was applicable to infected fish, taking into account effects of parasites. The uptake by parasites as well as the effects of parasites on the toxicokinetics of the fish host were taken into consideration while estimating metal accumulation in the host by the model. In such application, the uptake of metals by parasites can be considered another efflux pathway for the accumulation in the fish host. This assumption is supported by the uptake of the bile-metal complexes by the acanthocephalan as mentioned above. The absorption of metals from the host intestine has also been suggested to contribute to the accumulation of non-essential metals in *H*. *reliquens* [87]. The assumption is further substantiated by the findings by Hursky and Pietrock [85] on the accumulation of Se in the system of the rainbow trout *O*. *mykiss* and the nematode *R*. *acus*. In particular, these authors showed that the concentration of Se in the uninfected trout reached a steady state after 4-weeks of dietary exposure to Se whereas slower, but continuous, accumulation of this metal in the infected trout was observed during the 7-week exposure. Furthermore, this difference has been suggested to be related to the uptake of Se by the nematode for growth and metabolism.

However, the model developed in the present study might not cover all possible mechanisms of metal uptake in parasites. For instance, the increased demand in the fish host for production of immune cells, peptides, proteins, and molecules against parasite infection, which might contribute to lower concentrations of Se in the infected trout than in the uninfected trout [85], was not considered in our model. The lack of effects of waterborne Pb exposure on the accumulation in the acanthocephalans of essential metals such as Fe, Cu, and Zn at the background concentration of the tap water in the present study might indicate the demand of the acanthocephalans for these metals. The demand of parasites for essential metals might play an important factor in metal partitioning in fish-parasite systems, as supported by the difference in the accumulation of essential and of non-essential metals in nematodes [87]. The exclusion of the demand for essential metals in both parasites and the fish host might contribute to the slight underestimation while predicting the accumulation of the essential metals in the chub-acanthocephalan system in the present study. Another factor that might affect the accumulation of metals in parasites is the cuticle structure [87]. Mazhar et al. [87] suggested that *P*. *nemipteri* takes up toxic metals directly through the blood of the host. Another plausible pathway is through the surroundings water [87], which is not considered in our model for the acanthocephalan based on the assumption of the dominant availability of some metals in the host intestine for the intestinal parasites.

Considering the uptake by parasites as a pathway of elimination in the host, data on the uptake and elimination rate for infected fish are required for proper simulation of metal accumulation in the selected fish-parasite system. However, these data are very scarce. Therefore, some assumptions were used in model development as illustrated in the present study. In particular, the absorption efficiency and the elimination rate constant for the infected fish were parameterised relative to the corresponding value for the uninfected fish. The uncertainty and sensitivity analyses in the present study clearly indicate that this parameterisation represents one important source of uncertainty, which became obvious by variations of almost two orders of magnitude across the scenarios. In model parameterisation in the present study, the uptake rate of essential metals such as Cu, Fe, and Zn by parasites was assumed to be similar to that for Pb due to the lack of the required data. This assumption is a potential source of uncertainties as different trends in the accumulation in fish-parasite systems have been shown for essential and for non-essential metals as discussed above. Because of the lacking data for infected fish, a relationship between the metal concentration in the whole fish and the corresponding concentration in muscle was used for parameterising the uptake rate of parasites as described in the *Methods* section. Such a relationship might not hold in all conditions. For instance, Bevelhimer et al. [102] investigated the relationship between the concentrations in the whole fish and the concentrations in muscle for a variety of metals such as Cd, Cu, Pb, Ni, U, V, Zn, and Hg. These authors found relatively constant concentrations of Cd, Cu, Pb, Ni, Zn, U, and V in the whole fish with varying concentrations in muscle. This observation was related to the narrow range of metal concentrations in the study of Bevelhimer et al. [102]. Therefore, the observation demonstrated by Bevelhimer et al. [102] does not affect the relevance of our assumption to the experimental conditions in the present study.

Uncertainties from the parameterisation of uptake kinetics are also inherent in simulation of metal accumulation in the uninfected fish. The developed mechanistic model in which uptake and elimination rates were derived based on the covalent index and fish weight allows wide application and extrapolation to different metals and different fish species. However, the covalent index could explain less than 20% of the variations in the absorption efficiency as shown in the model development. This weak relationship between the covalent index and the absorption efficiency indicates that the absorption efficiency is not entirely metal-specific. Specifically, the absorption efficiency may depend on physiological characteristics of the organisms as well as physicochemical properties of the environment. For example, metal uptake might be enhanced due to the induction of metallothionein as a response to metal exposure [103]. The slight underestimation for essential metals such as Zn may be related to the exclusion of metallothionein induction, which could increase the uptake of the metals. Bervoets et al. [104] demonstrated the dependence of metallothionein induction by different fish species on exposure conditions. Environmental conditions were also shown to have influence on metal elimination by fish [105]. Metal uptake in fish is influenced by water chemistry where pH, salinity, dissolved organic carbon, and hardness play a significant role [8,41,42,55,106,107,108]. These aspects were not included in the present model, similar to mechanistic models developed previously [8,42]. The low potential of the covalent index for explaining the variations in the absorption efficiency leads to the question about the relevance of estimating this physiological rate constant by this approach while excluding physiological characteristics of organisms and physicochemical properties of the environment.

Results in the present study show the potential application of mechanistic models to simulating metal accumulation in fish and in fish-parasite systems as well. However, assumptions, which are required due to the lack of data, as well as the exclusion of some factors, which might influence metal uptake and elimination rates as mentioned above, represent potential sources of uncertainties. The model predictions can be improved by further calibration with more experimental data on metal uptake and elimination rates in parasites and in the fish host. In the present study, model validation was carried out based on limited data in our experimental investigation on a small number of fish due to ethical issues. Validation with a larger data set is required to obtain more comprehensive assessment on the performance of the developed model.

## Supporting Information

S1 Fig The dissolved concentrations of Fe, Cu, Zn, and Pb in the Pb exposure experiment for the uninfected (A) and infected (B) chub. The left Y axis represents the concentration of Fe, Cu, and Zn while the right Y axis represent the concentration of Pb in the tank water.
(TIF)

S2 Fig Relationship between ^210^Pb concentrations in muscle and the concentrations in the whole fish. The concentration in the whole fish was calculated from the concentrations and weights of gills, muscle, liver, intestine, and gallbladder, ignoring the negligible contribution of kidney and blood.
(TIF)

S3 Fig Relationship between the concentrations of Fe, Mn, Zn, Cu, Pb, Ni, and Cd in muscle and the corresponding concentrations in the whole fish analysed by Honda et al. [23]. (TIF)

S1 File Mathematical derivation of the metal concentration in the whole fish exposed to fluctuating exposure concentrations. (DOCX)

S2 File Parameterisation of the uptake rate of parasites. (DOCX)

S3 File References in Supporting Information. (DOCX)

S1 Table The recovery rates of Fe, Cu, Zn, and Pb in three reference materials: IAEA-407 (Fish Homogenate), DORM-2 (Dogfish Muscle Certified Reference Material), and DOLT-3 (Dogfish Liver Certified Reference Material) determined by the ICP-MS. (DOCX)

S2 Table Collected data on the dissolved uptake rate. (DOCX)

S3 Table Statistical parameters showing the relationship between the absorption efficiency and chemical properties of metals. (DOCX)

S4 Table Collected data on the elimination rate. (DOCX)

S5 Table Statistical parameters showing the relationship between the elimination rate and chemical properties of metals. (DOCX)
