# Supplementary figures and images for: Development and Validation of a Biodynamic Model for Mechanistically Predicting Metal Accumulation in Fish-Parasite Systems
Source: PLoS One. 2016 Aug 22;11(8):e0161091. doi: 10.1371/journal.pone.0161091 (PMC4993497; doi:10.1371/journal.pone.0161091)

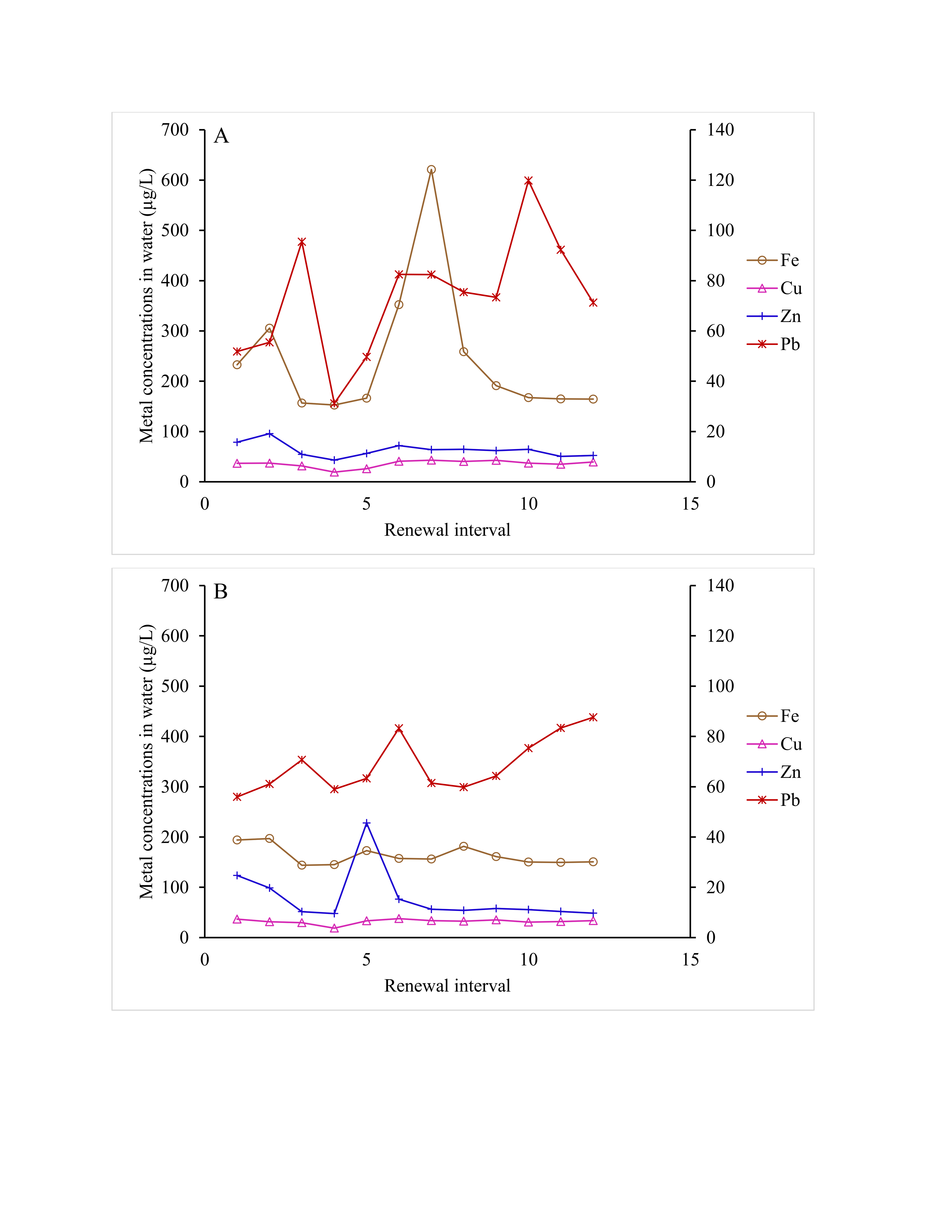

Supplement: S1 Fig — The left Y axis represents the concentration of Fe, Cu, and Zn while the right Y axis represent the concentration of Pb in the tank water. (TIF) [file pone.0161091.s001.tif]

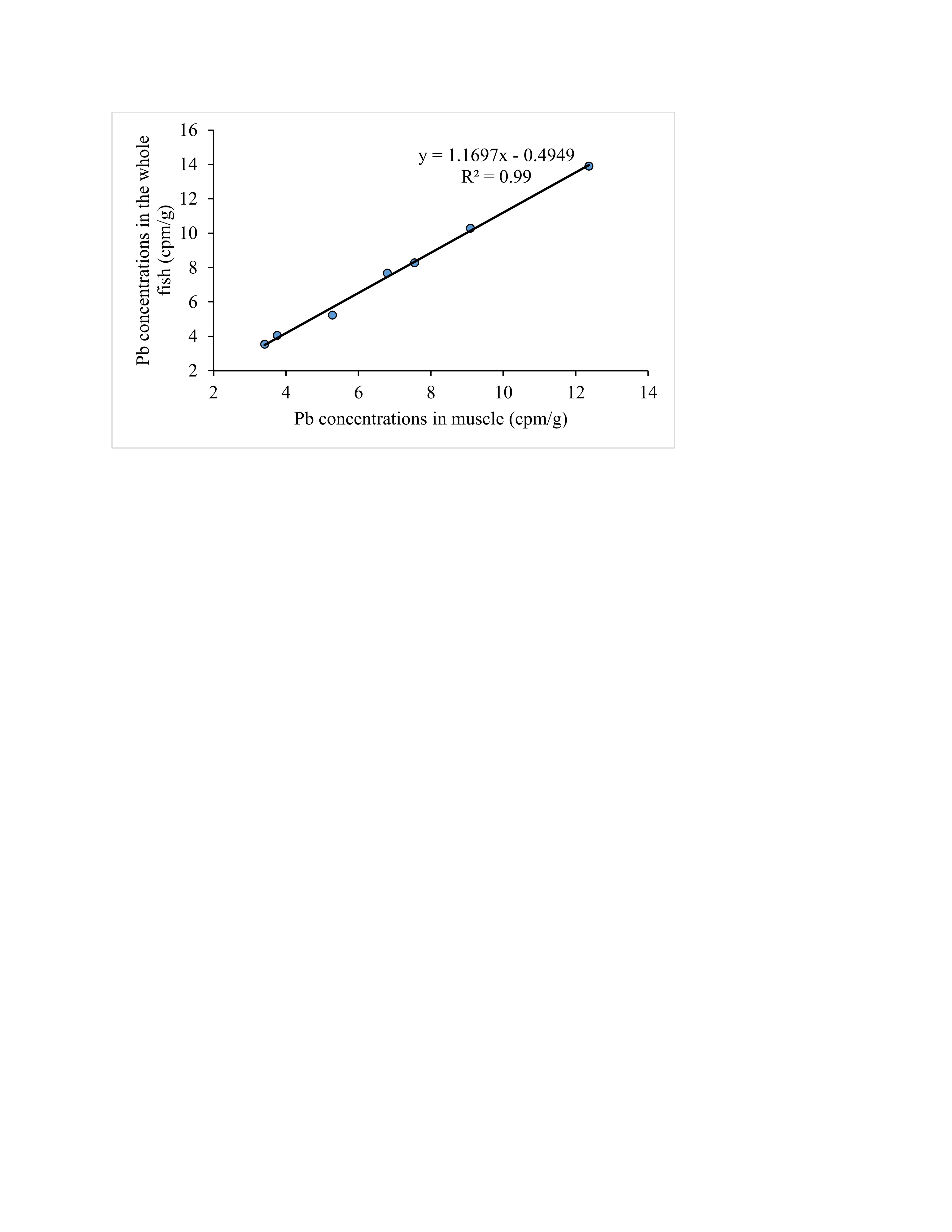

Supplement: S2 Fig — The concentration in the whole fish was calculated from the concentrations and weights of gills, muscle, liver, intestine, and gallbladder, ignoring the negligible contribution of kidney and blood. (TIF) [file pone.0161091.s002.tif]

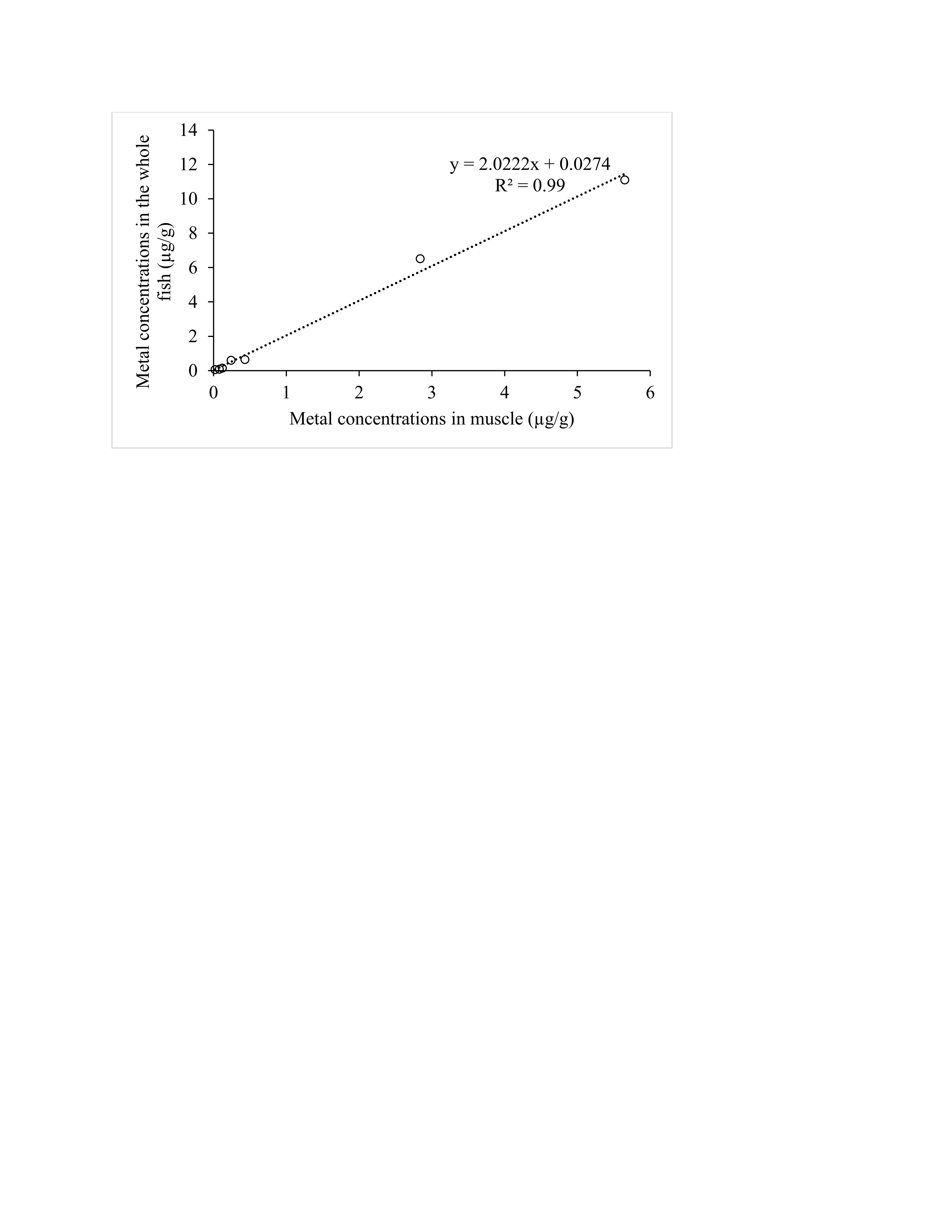

Supplement: S3 Fig — (TIF) [file pone.0161091.s003.tif]
