# Supplementary material for: Development and Validation of a Biodynamic Model for Mechanistically Predicting Metal Accumulation in Fish-Parasite Systems
Source: PLoS One. 2016 Aug 22;11(8):e0161091. doi: 10.1371/journal.pone.0161091 (PMC4993497; doi:10.1371/journal.pone.0161091)
Supplement: S1 File — (DOCX) [file pone.0161091.s004.docx]

**SA. Mathematical derivation of the metal concentration in the whole fish exposed to fluctuating exposure concentrations**

The changing in metal concentrations in fish can therefore be described by the following equation:

$\frac{\mathrm{dC}_{t}}{\mathrm{dt}}=k_{u}\times\mathrm{Cw}_{i}-(k_{e}+g)\times C_{t}$ (S1)

where C*_t_* (µg/g ww) is the metal concentration in the fish; *k*­_u­_ (L/g/d) is the dissolved uptake rate constant; Cw_i_ (µg/L) is the metal concentration in the exposure solution; *k*_e_ (1/d) is the elimination rate constant, excluding a distinction between different elimination pathways; and g (1/d) is the mass-based growth rate constant.

The accumulation of metals in fish exposed to changing exposure concentration was modelled by dividing the exposure duration into *n* intervals corresponding to each time of renewal (*T_j_*) and assuming that metal concentrations during the intervals were constant, as applied in previous studies [1,2]. At the first interval ($t\in\left[ T_{0}; T_{1} \right]$), the metal concentration in fish can be described as:

$C_{1}=C_{0}\times e^{-\left( k_{e}+g \right)\times\left( t-T_{0} \right)}+\frac{k_{u}}{k_{e}+g}\times\mathrm{Cw}_{1}\times\left( 1-e^{-\left( k_{e}+g \right)\times\left( t-T_{0} \right)} \right)$ (S2)

where C_0_ (µg/g ww) is the metal concentration in fish at the beginning of the exposure experiment; C_1_ (µg/kg ww) is the metal concentration in fish at a time before the first renewal; and Cw_1_ (µg/L) is the metal concentration in tank water before the first renewal [3,4].

The metal concentration in fish before the first renewal was considered the initial concentration in fish to model the fish concentration during the first and second renewal. Accordingly, metal concentrations in fish at the second interval ($t\in\left[ T_{1}; T_{2} \right]$) can be expressed by the following equation:

$C_{2}=\left[ C_{0}\times e^{-\left( k_{e}+g \right)\times\left( T_{1}-T_{0} \right)}+\frac{k_{u}}{k_{e}+g}\times\mathrm{Cw}_{1}\times\left( 1-e^{-\left( k_{e}+g \right)\times\left( T_{1}-T_{0} \right)} \right) \right]\times e^{-\left( k_{e}+g \right)\times\left( t-T_{1} \right)}+\frac{k_{u}}{k_{e}+g}\times\mathrm{Cw}_{2}\times\left( 1-e^{-\left( k_{e}+g \right)\times\left( t-T_{1} \right)} \right)$ (S3)

Equation S3 can be further elaborated as:

$C_{2}=C_{0}\times e^{-\left( k_{e}+g \right)\times\left( t-T_{0} \right)}+\frac{k_{u}}{k_{e}+g}\times\mathrm{Cw}_{1}\times\left( e^{-\left( k_{e}+g \right)\times\left( t-T_{1} \right)}-e^{-\left( k_{e}+g \right)\times\left( t-T_{0} \right)} \right)+\frac{k_{u}}{k_{e}+g}\times\mathrm{Cw}_{2}\times\left( 1-e^{-\left( k_{e}+g \right)\times\left( t-T_{1} \right)} \right)$ (S4)

$C_{2}=C_{0}\times e^{-\left( k_{e}+g \right)\times\left( t-T_{0} \right)}+\frac{k_{u}}{k_{e}+g}\times\left( \mathrm{Cw}_{1}\times\left( e^{-\left( k_{e}+g \right)\times\left( t-T_{1} \right)}-e^{-\left( k_{e}+g \right)\times\left( t-T_{0} \right)} \right)+\mathrm{Cw}_{2}\times\left( 1-e^{-\left( k_{e}+g \right)\times\left( t-T_{1} \right)} \right) \right)$ (S5)

The above equation can be generalised for any time *t* between the renewal *T_j_* and *T_j+1_*, yielding to an equation, similar to that developed by Adam et al. [1]:

$C_{t}=C_{0}\times e^{-\left( k_{e}+g \right)\times\left( t-T_{0} \right)}+\frac{k_{u}}{k_{e}+g}\times\left( \sum\mathrm{Cw}_{j}\times\left( e^{-\left( k_{e}+g \right)\times\left( t-T_{j} \right)}-e^{-\left( k_{e}+g \right)\times\left( t-T_{j-1} \right)} \right)+\mathrm{Cw}_{j+1}\times\left( 1-e^{-\left( k_{e}+g \right)\times\left( t-T_{j} \right)} \right) \right)$ (S6)
