# Supplementary material for: Development and Validation of a Biodynamic Model for Mechanistically Predicting Metal Accumulation in Fish-Parasite Systems
Source: PLoS One. 2016 Aug 22;11(8):e0161091. doi: 10.1371/journal.pone.0161091 (PMC4993497; doi:10.1371/journal.pone.0161091)
Supplement: S2 File — (DOCX) [file pone.0161091.s005.docx]

**S2. Parameterisation of the uptake rate of parasites**

Metal concentrations in parasites can be simulated by the following mass balance equation:

$\frac{\mathrm{dC}_{p}}{\mathrm{dt}}=k_{p}\times C_{t}\times\frac{W}{W_{p}}-n_{p}\times g_{p}\times C_{p}$ (S7)

where C_p_ (µg/kg ww) is the metal concentration parasites; *k*_p_ (1/d) is the uptake rate constant by parasites; W­­­ (g) is the whole fish weight; W_p_ (g) is the weight of parasites; *n*_p_ is the number of parasite individual in the fish host; and *g*_p_ (1/d) is the growth rate constant of parasites. This equation can be re-written as:

$\frac{\mathrm{dC}_{p}}{\mathrm{dt}}=k_{p}\times\mathrm{Cf}_{i}-n_{p}\times g_{p}\times C_{p}$ (S8)

where Cf_i_ (µg/kg wt) represents the fish source:

$\mathrm{Cf}_{i}=C_{t}\times\frac{W}{W_{p}}$ (S9)

where C*_t_* (µg/kg wt) is the metal concentration in fish; W (g) is the whole fish weight; and W_p_ (g) is the weight of parasites in the fish. Because of the insignificant changes in the weight of the whole fish (Table S6), the average of the fish collected at different sampling times were used for the above equation. Using this method, Cf_i_ was relatively constant (Fig S4 and Table S6). Consequently, ^210^Pb was considered to be taken from a constant source.

**Table A. The growth of chub in weight during a 35-day period**

| **Day** | **Concentrations in muscle (cpm/g)** | **Estimated concentrations in whole fish (cpm/g)** | **Whole fish weight W (g)** | **Total parasite weight Wp (g)** | **Cf_i_ (cpm/g)** |
| --- | --- | --- | --- | --- | --- |
| 0 | 5.28 | 5.69 | 9.6 | 4.38**^.^** 10^-3^ | 8528.19 |
| 3 | 3.41 | 3.49 | 10.0 | 7.60**^.^** 10^-3^ | 8265.48 |
| 10 | 7.55 | 8.33 | 11.6 | 8.10**^.^** 10^-3^ | 9682.58 |
| 17 | 3.76 | 3.90 | 12.8 | 6.72**^.^** 10^-3^ | 6090.44 |
| 24 | 6.80 | 7.45 | 10.2 | 1.60**^.^** 10^-2^ | 6169.36 |
| 31 | 9.09 | 10.14 | 9.7 | 1.30**^.^** 10^-2^ | 8892.27 |
| 38 | 12.36 | 13.96 | 9.8 | 1.34**^.^** 10^-2^ | 8892.27 |
| Average |  |  | 10.5 |  | 8074.37 |

**Fig A. The time-dependent source of ^210^Pb from the chub to parasites.** The black line represents the instant source estimated at sampling times and the orange line represents the average value.

Based on the assumption that the source of ^210^Pb in chub for the parasites was constant, ^210^Pb concentrations in the parasites could be expressed by the following equation:

$C_{p}=\frac{k_{p}}{n_{p}\times g_{p}}\times\mathrm{Cf}_{i}\times\left( 1-e^{-n_{p}\times g_{p}\times t} \right)$ (S10)

Subsequently, the uptake rate of ^210^Pb by parasites from the chub was determined based on Equation S10, i.e. by optimising the similarity between the ^210^Pb concentrations in parasites Cp estimated by Equation S4 and the concentrations measured by Sures et al. [24]. A value of 1.36^.^ 10^-3^ was determined for *k*_p_ through the non-linear regression analysis using the Sigma Plot. The time-dependent concentrations of ^210^Pb in parasites modelled and measures are given in Fig S5:

**Fig B. The time-dependent concentrations of ^210^Pb in parasites modelled (represented by the orange asterisk) and measured (represented by the line) in the study of Sures et al. [24]**
