# Supplementary material for: Development and Validation of a Biodynamic Model for Mechanistically Predicting Metal Accumulation in Fish-Parasite Systems
Source: PLoS One. 2016 Aug 22;11(8):e0161091. doi: 10.1371/journal.pone.0161091 (PMC4993497; doi:10.1371/journal.pone.0161091)
Supplement: S3 File — (DOCX) [file pone.0161091.s006.docx]

**References**

1. Adam C, Garnier-Laplace J, Baudin JP (1997) Uptake from water, release and tissue distribution of ^54^Mn in the rainbow trout (*Oncorhynchus mikiss* Walbaum). Environ Pollut 97: 29–38.
2. Garnier-Laplace J, Vray F, Baudin JP (1997) A dynamic model for radionuclide transfer from water to freshwater fish. Water Air Soil Pollut 98: 141–166.
3. Ciardullo S, Aureli F, Coni E, Guandalini E, Iosi F, Raggi A, Rufo G, Cubadda F (2008) Bioaccumulation potential of dietary arsenic, cadmium, lead, mercury, and selenium in organs and tissues of rainbow trout (*Oncorhyncus mykiss*) as a function of fish growth. J Agric Food Chem 56: 2442–2451.
4. Newman MC (2010) Fundamentals of Ecotoxicology. CRC Press, Boca Raton.
5. Long A, Wang W-X (2005) Assimilation and bioconcentration of Ag and Cd by the marine black bream after waterborne and dietary metal exposure. Environ Toxicol Chem 24: 709–716.
6. Pickhardt PC, Stepanova M, Fisher NS (2006) Contrasting uptake routes and tissue distributions of inorganic and methylmercury in mosquitofish (*Gambusia affinis*) and redear sunfish (*Lepomis microlophus*). Environ Toxicol Chem 25: 2132–2142.
7. Wang W-X, Kong RSK (2003) Bioaccumulation kinetics and exposure pathways of inorganic mercury and methylmercury in a marine fish, the sweetlips *Plectorhinchus gibbosus*. Mar Ecol Prog Ser 261: 257–268.
8. Pentreath (1973a) The accumulation from sea water of ^65^Zn, ^54^Mn, ^58^Co and ^59^Fe by the thornback ray, *Raja clavata* L. J Exp Mar Biol Ecol 12: 327–334.
9. Pentreath (1973b) The accumulation and retention of ^65^Zn and ^54^Mn by the plaice, *Pleuronectes platessa* L. J Exp Mar Biol Ecol 12: 1–18.
10. Pentreath (1973c) The accumulation and retention of ^59^Fe and ^58^Co by the plaice, *Pleuronectes platessa* L. J Exp Mar Biol Ecol 12: 315–326.
11. Pentreath (1976) The accumulation of inorganic mercury from sea water by the plaice, *Pleuronectes platessa* L. J Exp Mar Biol Ecol 24: 103–119.
12. Mathews T, Fisher NS, Jeffree RA, Teyssie J-L (2008) Assimilation and retention of metals in teleost and elasmobranch fishes following dietary exposure. Mar Ecol Prog Ser 360: 1–12.
13. Chu W-S, Wang J-P, Hou Y-Y, Ueng Y-T, Chu P-H (2011) Length-weight relationships for fishes off the southwestern coast of Taiwan. Afr J Biotechnol 10: 3945–3950.
14. Kulbicki M, Moutham G, Thollot P, Wantiez L. Length-weight relationships of fish from the lagoon of New Caledonia. Naga, the ICLARM Quarterly 16: 26–30.
15. Garnier-Laplace J, Adam C, Baudin JP (2000) Experimental kinetic rates of food-chain and waterborne radionuclide transfer to freshwater fish: a basis for the construction of fish contamination charts. Arch Environ Contam Toxicol 39: 133–144.
16. Jeffree RA, Warnau M, Teyssie J-L, Markich SJ (2006) Comparison of the bioaccumulation from seawater and depuration of heavy metals and radionuclides in the spotted dogfish *Scyliorhinus canicula* (Chondrichthys) and the turbot *Psetta maxima* (Actinoptegrygii: Teleostei). Sci Total Environ 368: 839–852.
17. Hogstrand C, Grosell M, Wood CM, Hansen H (2003) Internal redistribution of radiolabelled silver among tissues of rainbow trout (*Oncorhynchus mykiss*) and European eel (*Anguilla anguilla*): the influence of silver speciation. Aquat Toxicol 63: 139–157.
18. Baudin JP, Fritsch AF (1989) Relative contributions of food and water in the accumulation of ^60^Co by a freshwater fish. Wat Res 23: 817–823.
19. Wicklund A, Runn P (1988) Calcium effects on cadmium uptake, redistribution, and elimination in minnows, *Phoxinus phoxinus*, acclimated to different calcium concentrations. Aquat Toxicol 13: 109–122.
20. Wood CM, Grosell M, Hogstrand C, Hansen H (2002) Kinetics of radiolabelled silver uptake and depuration in the gills of rainbow trout (*Oncorhynchus mykiss*) and European eel (*Anguilla anguilla*): the influence of silver speciation. Aquat Toxicol 56: 197–213.
21. Xu Y, Wang W-X (2002) Exposure and potential food chain transfer factor of Cd, Se and Zn in marine fish *Lutjanus argentimaculatus*. Mar Ecol Prog Ser 238: 173–186.
22. Torres F JR (1991) Tabular data on marine fishes from Southern Africa, Part I: length-weight relationships. Fishbyte 9: 50–53.
23. Honda K, Sahrul M, Hidaka H, Tatsukawa R (1983) Organ and tissue distribution of heavy metals, and their growth-related changes in Antarctic fish, *Pagothenia borchgrevinki*. Agric Biol Chem 47: 2521–2532.
24. Sures B, Dezfuli BS, Krug HF (2003) The intestinal parasite *Pomphorhynchu laevis* (Acanthocephala) interferes with the uptake and accumulation of lead (^210^Pb) in its fish host chub (*Leuciscus cephalus*). Int J Parasitol 33: 1617–1622.
