# Supplementary material for: Development and Validation of a Biodynamic Model for Mechanistically Predicting Metal Accumulation in Fish-Parasite Systems
Source: PLoS One. 2016 Aug 22;11(8):e0161091. doi: 10.1371/journal.pone.0161091 (PMC4993497; doi:10.1371/journal.pone.0161091)
Supplement: S1 Table — (DOCX) [file pone.0161091.s007.docx]

**Table S1. The recovery rates of Fe, Cu, Zn, and Pb in three reference materials: IAEA-407 (Fish Homogenate), DORM-2 (Dogfish Muscle Certified Reference Material), and DOLT-3 (Dogfish Liver Certified Reference Material) determined by the ICP-MS**

| **Reference material** | **Fe** | **Cu** | **Zn** | **Pb** |
| --- | --- | --- | --- | --- |
| IAEA | 98.13 | 105.46 | 87.10 | 109.40 |
| DORM-2 | 77.55 | 99.94 | 86.01 | 90.68 |
| DOLT-3 | 82.54 | 109.53 | 96.41 | 85.27 |
