# Supplementary material for: Development and Validation of a Biodynamic Model for Mechanistically Predicting Metal Accumulation in Fish-Parasite Systems
Source: PLoS One. 2016 Aug 22;11(8):e0161091. doi: 10.1371/journal.pone.0161091 (PMC4993497; doi:10.1371/journal.pone.0161091)
Supplement: S2 Table — (DOCX) [file pone.0161091.s008.docx]

**Table S2. Collected data on the dissolved uptake rate**

| **Metal** | **Fish species** | **Fish weight (g)** | **Fish length (cm)** | **Absorption rate (L/kg/d)** | **References** |
| --- | --- | --- | --- | --- | --- |
| Ag | *Acanthopagrus schlegeli* | 1.02* | 3 | 17.43 | Long and Wang [5] |
| Cd | *Acanthopagrus schlegeli* | 1.02* | 3 | 4.68 | Long and Wang [5] |
| Hg | *Gambusia affinis;* | 0.45 |  | 65 | Pickardt et al. [6] |
| Hg | *Lepomis microlophus* | 0.9 |  | 44.5 | Pickardt et al. [6] |
| Hg | *Plectorhinchus gibbosus* | 2.04** | 3.25 | 195 | Wang and Wong [7] |
| Zn | *Raja clavata* | 35 |  | 14.74 | Pentreath [8] |
| Mn | *Raja clavata* | 35 |  | 22.60 | Pentreath [8] |
| Fe | *Raja clavata* | 35 |  | 84.04 | Pentreath [8] |
| Co | *Raja clavata* | 35 |  | 674.91 | Pentreath [8] |
| Zn | *Pleuronectes platessa* | 30 |  | 3.84 | Pentreath [9] |
| Mn | *Pleuronectes platessa* | 30 |  | 10.87 | Pentreath [9] |
| Fe | *Pleuronectes platessa* | 30 |  | 1.58 | Pentreath [10] |
| Co | *Pleuronectes platessa* | 30 |  | 55.45 | Pentreath [10] |
| Hg | *Pleuronectes platessa* | 42 |  | 4.3 | Pentreath [11] |
| Am | *Sparus auratus* | 12 |  | 10 | Mathews et al. [12] |
| Cd | *Sparus auratus* | 12 |  | 5 | Mathews et al. [12] |
| Co | *Sparus auratus* | 12 |  | 1 | Mathews et al. [12] |
| Cr | *Sparus auratus* | 12 |  | 1 | Mathews et al. [12] |
| Cs | *Sparus auratus* | 12 |  | 7 | Mathews et al. [12] |
| Mn | *Sparus auratus* | 12 |  | 2 | Mathews et al. [12] |
| Zn | *Sparus auratus* | 12 |  | 4 | Mathews et al. [12] |

*The fish weight was estimated from the weight-length relationship developed by Chu et al. [13]; **The fish weight was calculated from the weight-length relationship derived by Kulbicki et al. [14].
