# Supplementary material for: Development and Validation of a Biodynamic Model for Mechanistically Predicting Metal Accumulation in Fish-Parasite Systems
Source: PLoS One. 2016 Aug 22;11(8):e0161091. doi: 10.1371/journal.pone.0161091 (PMC4993497; doi:10.1371/journal.pone.0161091)
Supplement: S3 Table — (DOCX) [file pone.0161091.s009.docx]

**Table S3. Statistical parameters showing the relationship between the absorption efficiency and chemical properties of metals**

| **Statistical parameters** | **Ionic radius** | **Electronegativity** | **Covalent index** | **Softness index** | **Ionic index** | **LogK_OH_** |
| --- | --- | --- | --- | --- | --- | --- |
| *p* | 0.98 | 0.40 | 0.31 | 0.54 | 0.78 | 0.32 |
| R^2^ | 1.09**^.^** 10^-4^ | 0.10 | 0.15 | 0.07 | 0.01 | 0.14 |
