# Supplementary material for: Development and Validation of a Biodynamic Model for Mechanistically Predicting Metal Accumulation in Fish-Parasite Systems
Source: PLoS One. 2016 Aug 22;11(8):e0161091. doi: 10.1371/journal.pone.0161091 (PMC4993497; doi:10.1371/journal.pone.0161091)
Supplement: S4 Table — (DOCX) [file pone.0161091.s010.docx]

**Table S4. Collected data on the elimination rate**

| **Metal** | **Fish species** | **Fish weight (g)** | **Fish length (cm)** | **Elimination** | **References** |
| --- | --- | --- | --- | --- | --- |
| Mn | *Oncorhynchus mikiss* | 7.33 |  | 0.0176 | Adam et al. [1] |
| Cs | *Cyprinus carpio L.* | 2.17 |  | 0.00654 | Garnier-Laplace et al. [2] |
| Co | *Oncorhynchus mykiss* | 7.5 |  | 0.0372 | Garnier-Laplace et al. [15] |
| Cs | *Oncorhynchus mykiss* | 7.5 |  | 0.017 | Garnier-Laplace et al. [15] |
| Hg | *Gambusia affinis;* | 0.45 |  | 0.0315 | Pickardt et al. [6] |
| Hg | *Lepomis microlophus* | 0.9 |  | 0.0325 | Pickardt et al. [6] |
| Hg | *Plectorhinchus gibbosus* | 2.04* | 3.25 | 0.0287 | Wang and Wong [7] |
| Zn | *Raja clavata* | 35 |  | 0.033007 | Pentreath [8] |
| Mn | *Raja clavata* | 35 |  | 0.030137 | Pentreath [8] |
| Fe | *Raja clavata* | 35 |  | 0.043322 | Pentreath [8] |
| Co | *Raja clavata* | 35 |  | 0.036481 | Pentreath [8] |
| Zn | *Pleuronectes platessa* | 30 |  | 0.00235 | Pentreath [9] |
| Mn | *Pleuronectes platessa* | 30 |  | 0.00453 | Pentreath [9] |
| Fe | *Pleuronectes platessa* | 30 |  | 0.006601 | Pentreath [10] |
| Co | *Pleuronectes platessa* | 30 |  | 0.010664 | Pentreath [10] |
| Hg | *Pleuronectes platessa* | 42 |  | 0.005134 | Pentreath [11] |
| Am | *Sparus auratus* | 12 |  | 0.052 | Mathews et al. [12] |
| Cd | *Sparus auratus* | 12 |  | 0.065 | Mathews et al. [12] |
| Co | *Sparus auratus* | 12 |  | 0.049 | Mathews et al. [12] |
| Cr | *Sparus auratus* | 12 |  | 0.02 | Mathews et al. [12] |
| Cs | *Sparus auratus* | 12 |  | 0.057 | Mathews et al. [12] |
| Mn | *Sparus auratus* | 12 |  | 0.032 | Mathews et al. [12] |
| Zn | *Sparus auratus* | 12 |  | 0.037 | Mathews et al. [12] |
| Co | *Scyliorhinus canicula* | 6.1 |  | 0.015 | Jeffree et al. [16] |
| Cs | *Scyliorhinus canicula* | 6.1 |  | 0.014 | Jeffree et al. [16] |
| Am | *Scyliorhinus canicula* | 6.1 |  | 0.0011 | Jeffree et al. [16] |
| Cd | *Scyliorhinus canicula* | 6.1 |  | 0.0079 | Jeffree et al. [16] |
| Zn | *Scyliorhinus canicula* | 6.1 |  | 0.0096 | Jeffree et al. [16] |
| Mn | *Scyliorhinus canicula* | 6.1 |  | 0.025 | Jeffree et al. [16] |
| Cr | *Psetta maxima* | 6.7 |  | 0.0004 | Jeffree et al. [16] |
| Co | *Psetta maxima* | 6.7 |  | 0.0105 | Jeffree et al. [16] |
| Cs | *Psetta maxima* | 6.7 |  | 0.027 | Jeffree et al. [16] |
| Am | *Psetta maxima* | 6.7 |  | 0.0068 | Jeffree et al. [16] |
| Cd | *Psetta maxima* | 6.7 |  | 0.02 | Jeffree et al. [16] |
| Zn | *Psetta maxima* | 6.7 |  | 0.0066 | Jeffree et al. [16] |
| Mn | *Psetta maxima* | 6.7 |  | 0.0018 | Jeffree et al. [16] |
| Ag | *Anguilla anguilla* | 60 |  | 0.011 | Hogstrand et al. [17] |
| Co | *Cyprinus carpio* | 9.1 |  | 0.026 | Baudin and Fritsch [18] |
| Cd | *Phoxinus phoxinus* | 1.25 |  | 0.0078 | Wicklund and Runn [19] |
| Ag | *Oncorhynchus mykiss* | 25 |  | 0.0536 | Wood et al. [20] |
| Ag | *Oncorhynchus mykiss* | 60 |  | 0.0855 | Wood et al. [20] |
| Cd | *Lutjanus argentimaculatus* | 0.01** | 2.5 | 0.025 | Xu and Wang [21] |
| Se | *Lutjanus argentimaculatus* | 0.01** | 2.5 | 0.027 | Xu and Wang [21] |
| Zn | *Lutjanus argentimaculatus* | 0.01** | 2.5 | 0.015 | Xu and Wang [21] |

*The fish weight was estimated from the weight-length relationship derived by Kulbicki et al. [14]; **The fish weight was calculated from the weight-length relationship developed by Torres [22].

**Table S4. Collected data on the elimination rate**

| **Metal** | **Fish species** | **Fish weight (g)** | **Fish length (cm)** | **Elimination** | **References** |
| --- | --- | --- | --- | --- | --- |
| Mn | *Oncorhynchus mikiss* | 7.33 |  | 0.0176 | Adam et al. [1] |
| Cs | *Cyprinus carpio L.* | 2.17 |  | 0.00654 | Garnier-Laplace et al. [2] |
| Co | *Oncorhynchus mykiss* | 7.5 |  | 0.0372 | Garnier-Laplace et al. [15] |
| Cs | *Oncorhynchus mykiss* | 7.5 |  | 0.017 | Garnier-Laplace et al. [15] |
| Hg | *Gambusia affinis;* | 0.45 |  | 0.0315 | Pickardt et al. [6] |
| Hg | *Lepomis microlophus* | 0.9 |  | 0.0325 | Pickardt et al. [6] |
| Hg | *Plectorhinchus gibbosus* | 2.04* | 3.25 | 0.0287 | Wang and Wong [7] |
| Zn | *Raja clavata* | 35 |  | 0.033007 | Pentreath [8] |
| Mn | *Raja clavata* | 35 |  | 0.030137 | Pentreath [8] |
| Fe | *Raja clavata* | 35 |  | 0.043322 | Pentreath [8] |
| Co | *Raja clavata* | 35 |  | 0.036481 | Pentreath [8] |
| Zn | *Pleuronectes platessa* | 30 |  | 0.00235 | Pentreath [9] |
| Mn | *Pleuronectes platessa* | 30 |  | 0.00453 | Pentreath [9] |
| Fe | *Pleuronectes platessa* | 30 |  | 0.006601 | Pentreath [10] |
| Co | *Pleuronectes platessa* | 30 |  | 0.010664 | Pentreath [10] |
| Hg | *Pleuronectes platessa* | 42 |  | 0.005134 | Pentreath [11] |
| Am | *Sparus auratus* | 12 |  | 0.052 | Mathews et al. [12] |
| Cd | *Sparus auratus* | 12 |  | 0.065 | Mathews et al. [12] |
| Co | *Sparus auratus* | 12 |  | 0.049 | Mathews et al. [12] |
| Cr | *Sparus auratus* | 12 |  | 0.02 | Mathews et al. [12] |
| Cs | *Sparus auratus* | 12 |  | 0.057 | Mathews et al. [12] |
| Mn | *Sparus auratus* | 12 |  | 0.032 | Mathews et al. [12] |
| Zn | *Sparus auratus* | 12 |  | 0.037 | Mathews et al. [12] |
| Co | *Scyliorhinus canicula* | 6.1 |  | 0.015 | Jeffree et al. [16] |
| Cs | *Scyliorhinus canicula* | 6.1 |  | 0.014 | Jeffree et al. [16] |
| Am | *Scyliorhinus canicula* | 6.1 |  | 0.0011 | Jeffree et al. [16] |
| Cd | *Scyliorhinus canicula* | 6.1 |  | 0.0079 | Jeffree et al. [16] |
| Zn | *Scyliorhinus canicula* | 6.1 |  | 0.0096 | Jeffree et al. [16] |
| Mn | *Scyliorhinus canicula* | 6.1 |  | 0.025 | Jeffree et al. [16] |
| Cr | *Psetta maxima* | 6.7 |  | 0.0004 | Jeffree et al. [16] |
| Co | *Psetta maxima* | 6.7 |  | 0.0105 | Jeffree et al. [16] |
| Cs | *Psetta maxima* | 6.7 |  | 0.027 | Jeffree et al. [16] |
| Am | *Psetta maxima* | 6.7 |  | 0.0068 | Jeffree et al. [16] |
| Cd | *Psetta maxima* | 6.7 |  | 0.02 | Jeffree et al. [16] |
| Zn | *Psetta maxima* | 6.7 |  | 0.0066 | Jeffree et al. [16] |
| Mn | *Psetta maxima* | 6.7 |  | 0.0018 | Jeffree et al. [16] |
| Ag | *Anguilla anguilla* | 60 |  | 0.011 | Hogstrand et al. [17] |
| Co | *Cyprinus carpio* | 9.1 |  | 0.026 | Baudin and Fritsch [18] |
| Cd | *Phoxinus phoxinus* | 1.25 |  | 0.0078 | Wicklund and Runn [19] |
| Ag | *Oncorhynchus mykiss* | 25 |  | 0.0536 | Wood et al. [20] |
| Ag | *Oncorhynchus mykiss* | 60 |  | 0.0855 | Wood et al. [20] |
| Cd | *Lutjanus argentimaculatus* | 0.01** | 2.5 | 0.025 | Xu and Wang [21] |
| Se | *Lutjanus argentimaculatus* | 0.01** | 2.5 | 0.027 | Xu and Wang [21] |
| Zn | *Lutjanus argentimaculatus* | 0.01** | 2.5 | 0.015 | Xu and Wang [21] |

*The fish weight was estimated from the weight-length relationship derived by Kulbicki et al. [14]; **The fish weight was calculated from the weight-length relationship developed by Torres [22].
