# Supplementary material for: Development and Validation of a Biodynamic Model for Mechanistically Predicting Metal Accumulation in Fish-Parasite Systems
Source: PLoS One. 2016 Aug 22;11(8):e0161091. doi: 10.1371/journal.pone.0161091 (PMC4993497; doi:10.1371/journal.pone.0161091)
Supplement: S5 Table — (DOCX) [file pone.0161091.s011.docx]

**Table S5. Statistical parameters showing the relationship between the elimination rate and chemical properties of metals**

| **Statistical parameters** | **Ionic radius** | **Electronegativity** | **Covalent index** | **Softness index** | **Ionic index** | **LogK_OH_** |
| --- | --- | --- | --- | --- | --- | --- |
| *p* | 0.16 | 0.38 | 0.07 | 0.84 | 0.29 | 0.52 |
| R^2^ | 0.20 | 0.09 | 0.32 | 0.01 | 0.12 | 0.05 |
